# The Complete Genome Sequence of Plodia Interpunctella Granulovirus: Evidence for Horizontal Gene Transfer and Discovery of an Unusual Inhibitor-of-Apoptosis Gene

**DOI:** 10.1371/journal.pone.0160389

**Published:** 2016-07-29

**Authors:** Robert L. Harrison, Daniel L. Rowley, C. Joel Funk

**Affiliations:** 1 Invasive Insect Biocontrol and Behavior Laboratory, Beltsville Agricultural Research Center, USDA Agricultural Research Service, Beltsville, Maryland, United States of America; 2 Department of Biology, John Brown University, Siloam Springs, Arkansas, United States of America; Ecole des Mines d'Alès, FRANCE

## Abstract

The Indianmeal moth, *Plodia interpunctella* (Lepidoptera: Pyralidae), is a common pest of stored goods with a worldwide distribution. The complete genome sequence for a larval pathogen of this moth, the baculovirus Plodia interpunctella granulovirus (PiGV), was determined by next-generation sequencing. The PiGV genome was found to be 112, 536 bp in length with a 44.2% G+C nucleotide distribution. A total of 123 open reading frames (ORFs) and seven homologous regions (*hr*s) were identified and annotated. Phylogenetic inference using concatenated alignments of 36 baculovirus core genes placed PiGV in the “b” clade of viruses from genus *Betabaculovirus* with a branch length suggesting that PiGV represents a distinct betabaculovirus species. In addition to the baculovirus core genes and orthologues of other genes found in other betabaculovirus genomes, the PiGV genome sequence contained orthologues of the bidensovirus NS3 gene, as well as ORFs that occur in alphabaculoviruses but not betabaculoviruses. While PiGV contained an orthologue of *inhibitor of apoptosis-5* (*iap-5*), an orthologue of *inhibitor of apoptosis-3* (*iap-3*) was not present. Instead, the PiGV sequence contained an ORF (PiGV ORF81) encoding an IAP homologue with sequence similarity to insect cellular IAPs, but not to viral IAPs. Phylogenetic analysis of baculovirus and insect IAP amino acid sequences suggested that the baculovirus IAP-3 genes and the PiGV ORF81 IAP homologue represent different lineages arising from more than one acquisition event. The presence of genes from other sources in the PiGV genome highlights the extent to which baculovirus gene content is shaped by horizontal gene transfer.

## Introduction

Viruses of family *Baculoviridae* infect insects of orders Lepidoptera, Diptera, and Hymenoptera [1, 2]. These viruses are characterized as having relatively large (80–180 kbp), circular, double-stranded DNA genomes that are packaged into rod-shaped, cylindrical capsids, which in turn are surrounded by a lipid envelope. Another key feature of these viruses is the formation of distinctive occlusion bodies consisting of a single highly-expressed viral protein (polyhedrin or granulin) assembled into a protective paracrystalline matrix around the enveloped virions.

Baculoviruses of genera *Alphabaculovirus* and *Betabaculovirus*, identified from moth and butterfly larvae, have received the most attention from researchers. Alphabaculovirus occlusion bodies (also referred to as polyhedra) are large polyhedral-shaped structures (0.15 to 15 μm) that contain multiple virions, often with >1 nucleocapsid per unit envelope [2]. Alphabaculoviruses, also known as nucleopolyhedroviruses (NPVs), can also productively infect insect cell lines derived from their hosts. This feature has facilitated much basic research on NPVs as well as the development of selected NPVs (such as Autographa californica multiple nucleopolyhedrovirus, or AcMNPV) into expression vectors [3]. Betabaculoviruses, also known as granuloviruses (GVs), differ by producing significantly smaller ovocylindrical occlusion bodies (approximately 0.13 x 0.5 μm) that contain a single virion [2]. There have been far fewer reports of successful infection of a cell line by a GV [4], and research with GVs has lagged behind that of NPVs.

A granulovirus reported to infect larvae of the Indianmeal moth, *Plodia interpunctella*, was first described in 1968 [5]. Study of this virus, Plodia interpunctella granulovirus (PiGV), led to the creation and recognition of the species *Plodia interpunctella granulosis virus* (later renamed as *Plodia interpunctella granulovirus*) in family *Baculoviridae* in 1982 [6].

*P*. *interpunctella* is a prevalent and cosmopolitan pest of stored grains, nuts, and other dried foodstuffs. As a consequence, research on PiGV was carried out to evaluate it as a potential alternative to chemical insecticides for controlling infestations of *P*. *interpunctella* [7–10]. This research culminated in the registration of PiGV as a biopesticide [11]. PiGV was also the subject of study in early research on the molecular biology of baculovirus structural proteins and virion assembly [12–14]. Finally, PiGV and *P*. *interpunctella* have been developed into a model system for understanding the ecology and evolution of infectious disease, particularly with respect to factors affecting pathogen transmission [15–18], host susceptibility [19–22], host dynamics [23, 24], and response to infection [25–27].

As of this writing, complete or partial DNA sequence data for > 50 betabaculovirus isolates have been deposited in GenBank, including the open reading frame (ORF) of a PiGV chitinase gene (GenBank accession no. KP864638). To fully characterize the genome of PiGV, we isolated and sequenced genomic DNA isolated from PiGV granules. The complete PiGV genome sequence was determined, and analysis of this sequence suggests that the species *Plodia interpunctella granulovirus* is distinct from other species of *Betabaculovirus*. Our analysis highlighted the remarkable degree to which this virus has acquired genes from other sources.

## Materials and Methods

### Virus DNA isolation

PiGV stocks were obtained from the USDA-ARS Manhattan, KS laboratory where PiGV biopesticide and molecular biology research took place during the 1970s and 1980s [28, 29]. This PiGV isolate was obtained from D. K. Hunter (USDA-ARS, Fresno, CA) in 1972, and originated with the isolate characterized by Arnott and Smith [5]. The virus was propagated in *P*. *interpunctella* larvae, separated from cell debris using a sucrose cushion, then purified using sucrose gradients [30]. Purified PiGV granules were solubilized in 0.1 M sodium carbonate and DNA was extracted from occluded virus as previously described [31, 32].

### Genome sequencing and assembly

Genomic DNA was sequenced as previously described [32] except that the DNA was fragmented and the MID tags added at University of Florida Interdisciplinary Center for Biotechnology Research (Gainesville, FL). Sequencing reads from a Roche 454 GS Junior instrument were sorted and assembled using the SeqMan NGEN V3.0 assembler program (Lasergene; DNASTAR, Inc., Madison, WI) with default parameters. PCR and Sanger dideoxy sequencing were carried out to close gaps in the alignment and resolve or confirm regions with ambiguous sequences or unusual features, including 17 N_8+_ homopolymer regions. The Lasergene SeqManPro (version 12) sequence editor was used to prepare the final contig of the consensus genome sequence. The PiGV genome sequence generated for this study has been deposited in GenBank with the accession number KX151395.

### ORF and homologous repeat region (*hr*) annotation

ORFs identified in the PiGV genome sequence were annotated if they were >50 codons in length and were evolutionarily conserved with other viral or non-viral ORFs, as ascertained by BLASTp. ORFs which did not yield a significant match by BLASTp (e.g. no matches with e-values <0.010) were annotated if they did not overlap a larger ORF by >75 bp and if they were predicted to be protein-encoding by both the fgenesV (http://linux1.softberry.com/berry.phtml) and ZCURVE_V [33] algorithms. In accordance with the convention for numbering baculovirus ORFs, the ORF encoding granulin was designated as ORF1, and the adenine of the granulin ORF start codon was designated as nt 1 of the genome.

Homologous region (*hr*) sequences were identified using the pattern- and repeat-finding functions of the LaserGene GeneQuest program (DNASTAR), Tandem Repeats Finder [34], and REPuter [35]. Individual repeats from the *hr*s were aligned using Clustal W in LaserGene MegAlign (DNASTAR), and the alignment and repeat consensus sequence were displayed with BOXSHADE (http://www.ch.embnet.org/software/BOX_form.html).

### Sequence comparison and phylogeny

Shared synteny between PiGV and Plutella xylostella granulovirus K1 (PlxyGV-K1) [36], Cydia pomonella granulovirus isolate M1 (CpGV-M1; [37], and Pieris rapae granulovirus isolate Wuhan (PiraGV-Wuhan; [38] was determined by constructing gene parity plots [39].

To identify genetic differences between the PiGV genome reported in this study and the PiGV transcriptome sequences reported by [40], pairwise alignment was carried out between the PiGV genome sequence and the PiGV transcriptome contigs located at http://afterparty.bio.ed.ac.uk/study/show/2194070 [40].

The relationship of PiGV to other baculoviruses was inferred from the concatenated alignments of 36 of 37 putative baculovirus core genes [41]. An alignment of P6.9 amino acid sequences was not included in the analysis, as ORF31 of the gammabaculovirus Neodiprion abietis nucleopolyhedrovirus (NeabNPV) does not encode a *p6*.*9* homologue and no complete *p6*.*9* ORF has not been identified in the NeabNPV genome. In addition, the homologue of the *ac78* core gene in NeabNPV is not ORF46 as reported by Garavaglia et al., but is instead an unannotated ORF extending from nt 42344←42628 in the NeabNPV genome. The amino acid sequence specified by this unannotated ORF was used in the AC78 alignment. The baculovirus core gene amino acid alignments were concatenated using BioEdit 7.1.3 [42].

In addition, amino acid sequence alignments were assembled with additional individual PiGV ORFs of interest (PiGV ORF11, ORF15, ORF18, and ORF81) and related viral and non-viral homologues of these ORFs.

In all cases, amino acid sequences were aligned by Clustal W using LaserGene MegAlign (DNASTAR) with default parameters, except for desmoplakin (AC66), AC78, PiGV ORF15, ORF18, and ORF81. For these alignments, the multiple and pairwise alignment penalties were reduced from 10 to 5 and the multiple alignment gap length penalty was reduced from 0.2 to 0.1 to compensate for the lower degree of conservation among the sequences of these proteins. Phylogenetic trees were constructed with the minimum evolution (ME) method using MEGA6 [43] with 500 bootstrap replicates. A phylogenetic tree was also inferred from the same alignment by maximum likelihood using either MEGA6 (for the individual PiGV ORF alignments) or RAxML 7.7.1 [44] (for the concatenated core gene alignment). MEGA6 was used to identify the best-fitting evolutionary models to use with these analyses and to calculate the gamma shape parameter for site rate differences.

The sequences used for phylogenetic inference are listed in S1 Table.

## Results and Discussion

### Properties of the PiGV genome

**Fig 1:**
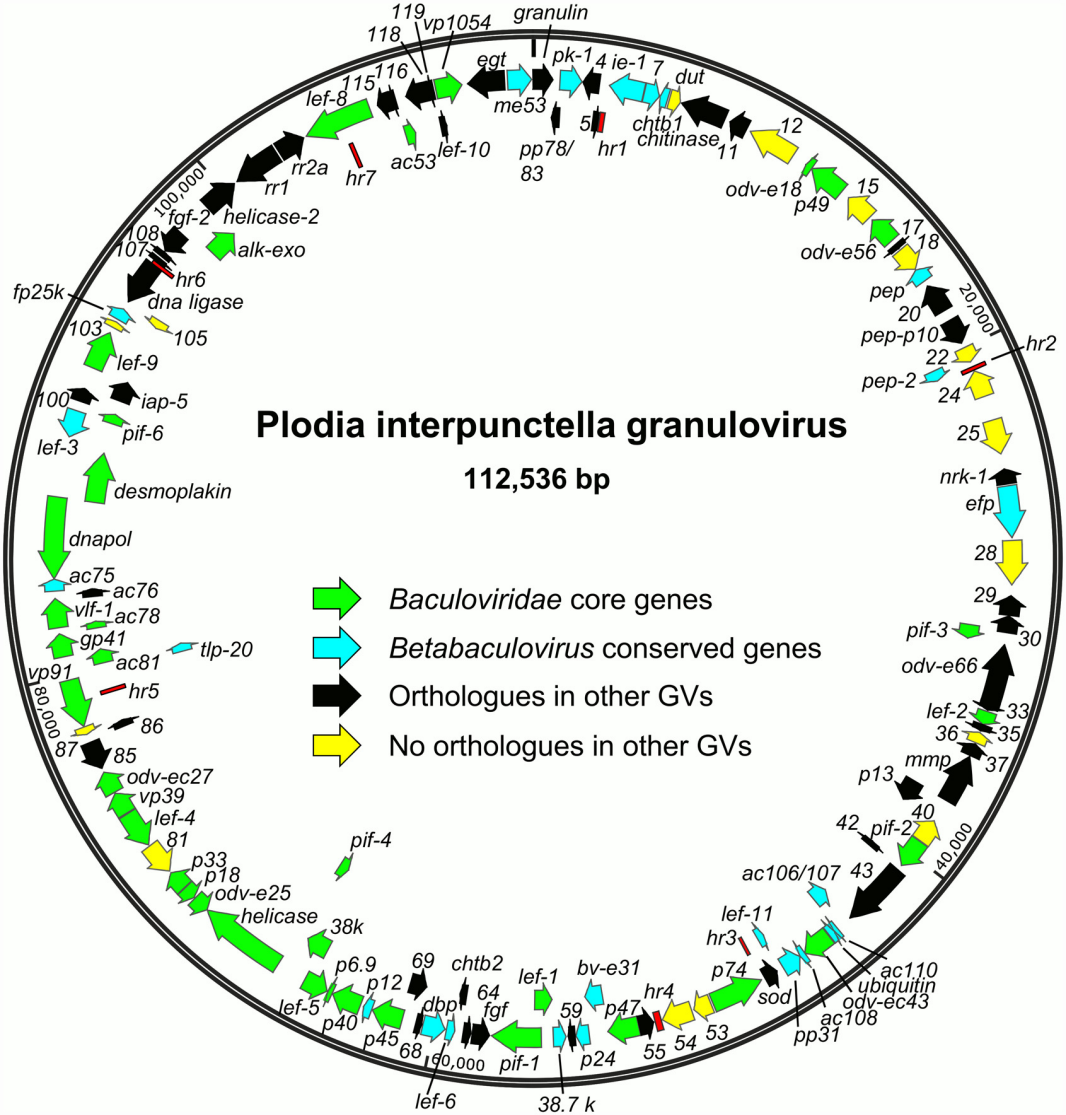
Map of the ORFs and other features of the PiGV genome. ORFs are represented by arrows, with the position and direction of the arrow indicating ORF position and orientation. Each ORF is designated by a number or, in the case of conserved or well-characterized baculovirus orthologues, a name. Categories of ORFs are indicated in the figure. Homologous repeat regions (*hr*s) are represented by red boxes.

Assembly of 454 and Sanger dideoxy sequencing data yielded a genome of 112, 536 bp with a coverage of 53.9 X (Fig 1). The size is lower than the mean GV genome size of 123,464 (n = 18), though this average is skewed by the inclusion of the unusually large Helicoverpa armigera GV (HearGV) and Xestia c-nigrum GV (XecnGV) genomes [45, 46]. The PiGV genome is closest in size to that of Cryptophebia leucotreta GV (CrleGV), which is 110, 907 bp [47]. The PiGV nucleotide distribution, at 44.2% G+C, is closest to that of Clostera anachoreta granulovirus-HBHN (ClanGV-HBHN; [48]) at 44.4% G+C. Up to 123 ORFs were annotated for the PiGV genome, with 64 in the sense direction and 59 in the antisense direction (Fig 1, S2 Table).

**Fig 2:**
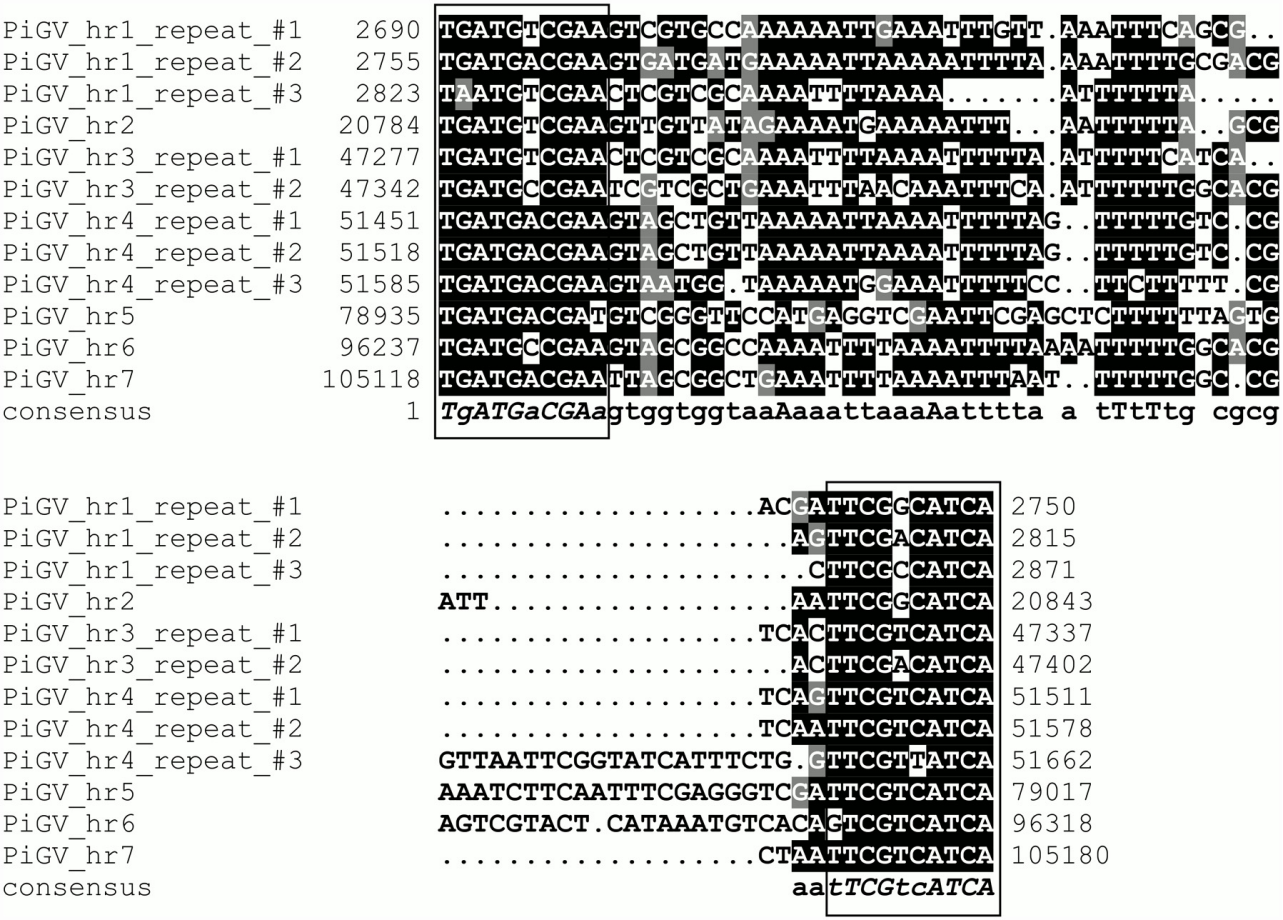
Alignment of PiGV homologous region (*hr*) palindromic repeats. Nucleotide positions of the repeats in the genome sequence are indicated. Identical nucleotides occupying >50% of aligned positions are shaded in black, and nucleotides of the same class as conserved nucleotides (containing either a purine or pyrimidine base) are shaded in gray. Nucleotides in the repeat consensus sequence are denoted by uppercase letters for positions in the alignment with completely identical residues, and lowercase letters for positions in the alignment with a majority of identical residues. The conserved 10-bp inverted terminal repeat sequence is indicated by boxes surrounding the aligned sequence at each end.

Seven homologous repeat regions (*hr*s) were identified in the PiGV genome. These *hr*s consist of 1–3 imperfect palindromes ranging in size from 49 to 83 bp and bound by inverted repeats with the consensus sequence TGATGACGAA (Fig 2). The consensus sequence of the PiGV *hr* terminal repeats did not closely match the consensus terminal sequence found to be conserved among the *hr*s of five GVs [49]. However, six of the seven PiGV *hr*s are located in positions that are conserved among other GV genomes for *hr*s, such as next to CpGV ORF *cp5* (*hr1*), between *sod* and *p74* (*hr3*), upstream of *p47* (*hr4*), within *vp91* (*hr5*), next to *dna ligase* (*hr6*), and within *lef-8* (*hr7*) [45]; Fig 1, S2 Table). Some *hr*s of CpGV and CrleGV have been found to act as origins of DNA replication in *C*. *pomonella* tissue culture-based assays [49, 50], and it seems likely that PiGV *hr*s perform the same function in PiGV-infected *P*. *interpunctella* cells.

### Relationships to other baculoviruses

Blastp queries with the conceptual amino acid sequences of PiGV ORFs yielded the highest-scoring matches with ORFs from a wide variety of betabaculoviruses, including isolates of CpGV, CrleGV, Agrotis segetum granulovirus (AgseGV), Choristoneura occidentalis granulovirus (ChocGV), Clostera anastomosis granulovirus-A (ClanGV-A, or CaLGV) and -B (ClanGV-B), Lacanobia oleracea granulovirus (LaolGV), Pieris rapae granulovirus (PiraGV), Phthorimaea operculella granulovirus (PhopGV), Epinotia aporema granulovirus (EpapGV), Erinnyis ello granulovirus (ErelGV), Spodoptera frugiperda granulovirus (SpfrGV), and Spodoptera litura granulovirus (SpltGV) (S2 Table). PiGV ORF1, encoding granulin, was the most conserved gene, exhibiting a 94.4% amino acid sequence similarity with the encoded granulin of CrleGV. Other ORFs encoding granulovirus homologues exhibited amino acid similarities ranging from 28.4% (PiGV ORF38, *mmp*) to 89.7% (PiGV ORF46, *ubiquitin*), with an average amino acid similarity of 54.8%.

**Fig 3:**
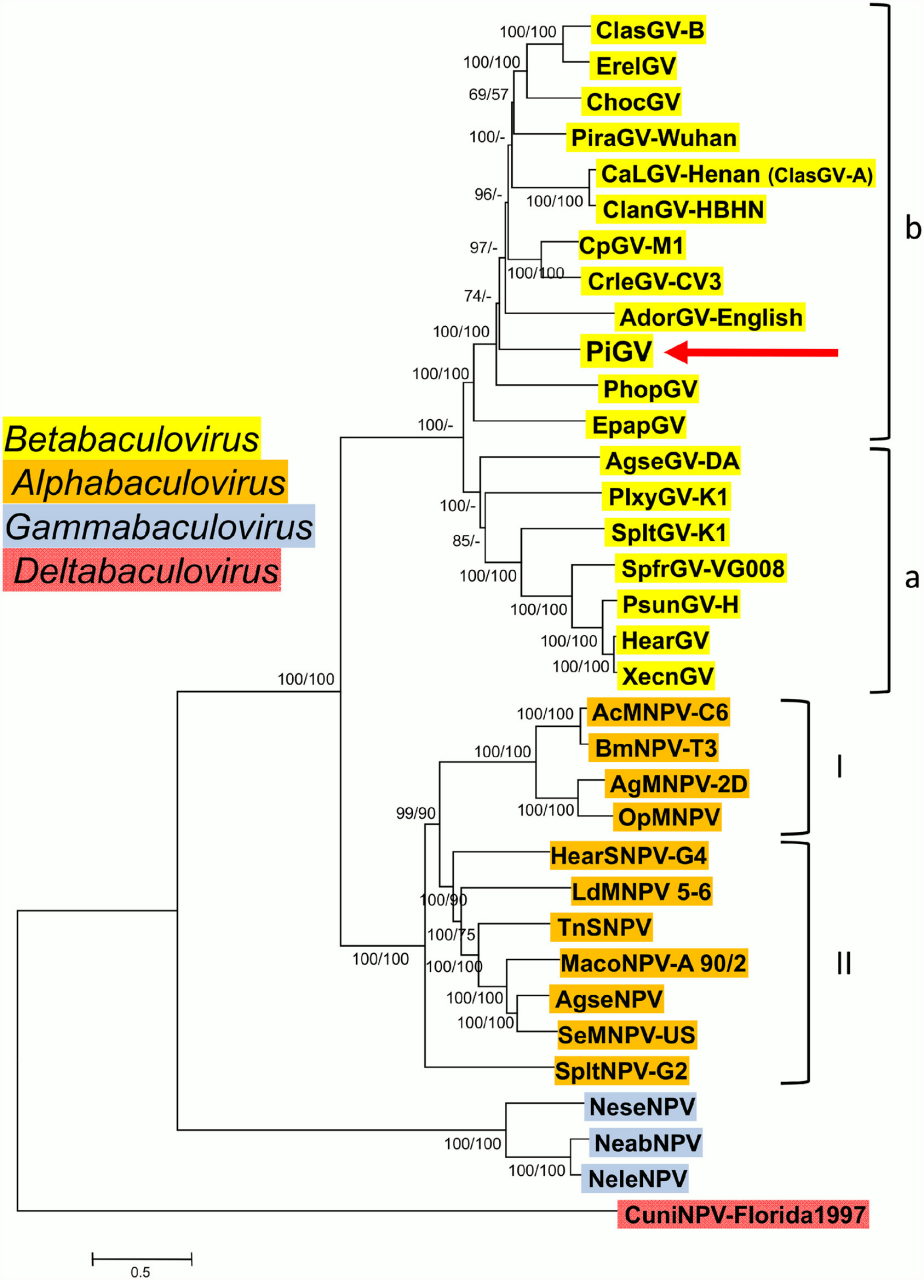
Relationships of PiGV and representative isolates of other baculovirus species, inferred from the predicted amino acid sequences of baculovirus core genes. The phylogenetic tree was constructed from the concatenated alignments of 36 baculovirus core gene amino acid sequences using the minimum-evolution (ME) method. Taxon genera are indicated with colored text background. Both the group I and II clades of genus *Alphabaculovirus* and the a and b clades of *Betabaculovirus* are indicated with brackets. Bootstrap values >50% for both ME and maximum likelihood (ML) analysis are indicated for each interior branch (ME/ML). In addition to PiGV (indicated by a red arrow), virus taxa and accession numbers used in the analysis are as indicated in S1 Table.

Phylogenetic inference with concatenated amino acid sequence alignments of 36 of the 37 currently accepted baculovirus core genes [41] confirmed that PiGV is a virus of genus *Betabaculovirus* (Fig 3). Miele and co-workers [51] proposed that betabaculoviruses occurred in two distinct groups, clade a and clade b. The ME phylogenetic tree (Fig 3) supports this division, but inference with ML placed PlxyGV outside of these two clades (data not shown). PiGV occurs in clade b, the support for its position among the other clade b granuloviruses is relatively weak with ME/ML bootstrap values of 74/-. The PiGV core gene concatemer is separated from other clade b sequences by long branch lengths and does not appear to be closely related to any particular GV.

**Fig 4:**
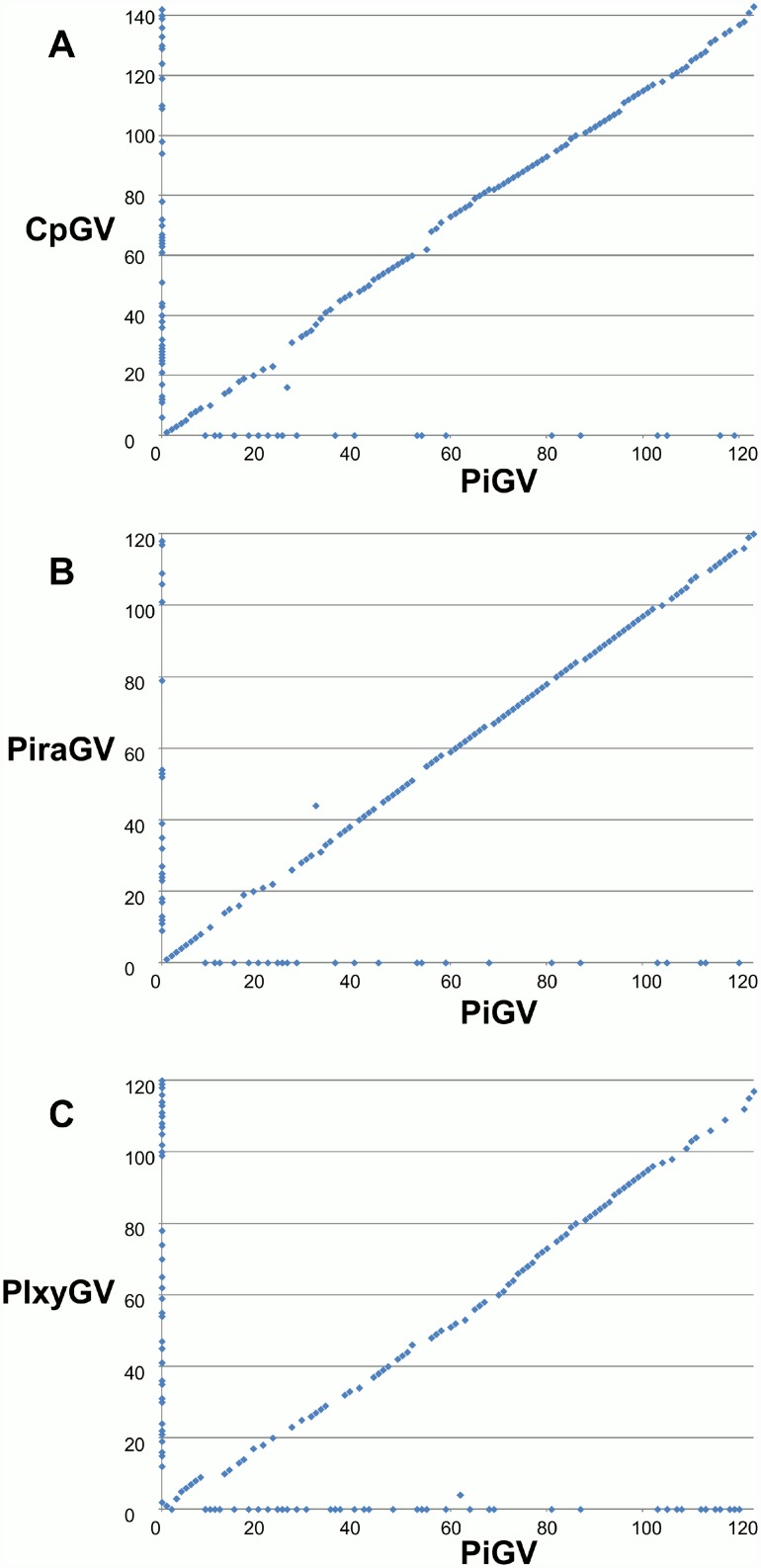
Gene parity plots comparing PiGV with representative clade a and b granuloviruses. Plots show the ORF content and order of the PiGV genome (x-axis) with that of (A) CpGV, (B) PiraGV, and (C) PlxyGV (y-axes). Each point in the plot represents an ORF. ORFs present in only one of the compared genomes appear on the axis corresponding to the virus in which they are present.

Gene parity plot analysis was carried out to visualize the synteny between the genomes of PiGV and two other clade b granuloviruses (CpGV-M1 and PiraGV-Wuhan). Gene order in the PiGV genome was also compared with that of a clade a granulovirus that has a genome of a comparable size (PlxyGV; 100,999 bp). Although the gene content differences between PiGV and PlxyGV were visibly more numerous than those between PiGV and the clade b GVs, the degree of synteny was not noticeably different (Fig 4).

Recently, the transcriptome of *P*. *interpunctella* larvae infected with PiGV and harvested at 24 hours post-infection (hr p. i.) was reported [40]. The authors of this study identified contigs of granulovirus origin (ascertained by BLAST alignment with the sequence of PlxyGV) and concluded that they had assembled viral sequence data from the infected *P*. *interpunctella* transcriptome amounting to an estimated 44% of the PiGV genome. Comparison of the PiGV genome with the viral contigs assembled from transcriptomic data [40] revealed that the virus used by McTaggart and co-workers is the same virus reported here. Transcriptome reads from this project assembled into eleven contigs that aligned with PiGV nt 2841–7436, 10664–13710, 18056–27589, 29803–34735, 64189–69653, 70348–71408, 72086–77471, and 79003–96283, for a total of 51, 303 bp or 45.6% of the genome. Two of the transcriptome contigs consist of an inverted repeat of the same sequence–for example, nt 1→ 4891 of contig comp66738_c0_seq1 aligns with the antisense strand of PiGV genome nt 88851←93740, while nt 4808→9698 of the same contig aligns with the sense strand of the same region of PiGV (nt 88851→93740). PCR with PiGV genomic DNA and primers that annealed close to the inversion points in these contigs failed to produce amplimers, indicating that the inversions in these contigs were the result of erroneous assembly. These contigs may have led McTaggart and co-workers to conclude that there were inversions of the PiGV genome sequence relative to that of PlxyGV in a dot-plot of the PiGV-derived sequence vs. the PlxyGV genome sequence, [40]. Our gene parity plot analysis does not support the presence of large-scale inversions in the PiGV genome relative to the genome of PlxyGV.

Alignment of the consensus PiGV genome and the McTaggart et al. viral transcriptomic sequences revealed a total of 66 substitutions (28 synonymous, 26 nonsymonymous, and 12 intergenic), for a rate of 1.3 substitutions/kbp (Table 1). In addition, 23 indels (12 intergenic and 11 located within ORFs) were detected. Thirty-six of the substitutions and six of the indels in Table 1 also were found in a minority of the 454 genome sequence reads, ranging in frequency from 10% (indel at nt 12508) to 48.8% (C to T substitution at nt 90715) with an average frequency of 27.9%. Of the 11 indels in ORFs, 10 of them were in-frame and 1 resulted in a frameshift within PiGV ORF14. This ORF is an orthologue of *p49* (AcMNPV ORF *ac142*), which appears to be required for virus production [52, 53]. The indel in *p49*, which increases a homopolymer repeat from A_8_ to A_9_, is present in 10% of the 454 genome sequence reads, suggesting that it corresponds to a minor genotype that may be dependent on P49 supplied in *trans*.

**Table 1:** Genetic variation between the consensus PiGV genome sequence and *P*. *interpunctella* transcriptome sequences of viral origin reported by McTaggart et al. [40].

Substitution or indel^a^	Gene or region affected
A5697G	Chitinase (ORF10); synonmous substitution
***G11428A***; G11902T; ***G12346A (A) inserted after 12508 (8A->9A)***	*p49* (ORF14); frameshift in 7^th^ codon, 2 synonymous substitutions and 1 non-synonymous substitutions occurring downstream.
Δ12640–12750; Δ12790–12792	Between ORF14 and ORF15
***T13000C***; G13487A; A13624G	ORF15; 3 synonymous substitutions
Δ18562–18635; Δ18777 (4T->3T)	Between ORF20 and ORF21
C18910A; C19166A; G19216A; A19248G; ***A19307G***; ***(GGT) inserted after 19336***; ***A19367G***; ***(CTTCAGACG) inserted after 19495***	*pep-p10* (ORF21); 4 nonsynonymous substitutions, 2 synonymous substitutions, 1 3 nt insertion, 1 9 nt insertion
Δ19809; ***T19960G***; T19963G	Between ORF21 and ORF22
***Δ20106–20114***	ORF22; 1 9-nt deletion
A21721T	ORF24; nonsynonymous substitution
T22038C; ***Δ22215–22224***; Δ22250; Δ22412; ***A22788G***	Between ORF24 and ORF25
Δ23361–23363; G23412A; ***(AACTTTCCAGCT) inserted after 23523***; (CGTCGACGAGCC) inserted after 23594; A23649G; Δ23669–23692 A23708G; T23738A; (AAAAAG) inserted after 23786	ORF25; 1 3-nt deletion, 1 24-nt deletion, 2 12-nt insertions, 1 6-nt insertion, 2 nonsynonymous substitutions, 2 synonymous substitutions
Δ24679 (9A to 8A); (T) inserted after 24699 (9T to 10T)	Between ORF25 and ORF26
C25274T	*nrk-1* (ORF26); nonsynonymous substitution
***C27052T***	*efp* (ORF27); synonymous substitution
C30502T	ORF30; synonymous substitution
***C31352T***	*pif-3* (ORF31); synonymous substitution
***G31427A***; ***G31440A***; G32000T; G32183A;C32591T; Δ33295–33357	*odv-e66* (ORF32); 1 63-nt deletion, 3 nonsynonymous substitutions, 2 synonymous substitutions
***C33677T***	ORF33; 1 synonymous substitution
G33929A; ***A33939G***	Between ORF33 and ORF34
***A34556G***; ***T34578C***; ***A34579C***; ***G34689T***	ORF35; 3 nonsynonymous substitutions and 1 synonymous substitution
***C64930T***; ***G65260C***	*38k* (ORF75); 2 synonymous substitutions
***C68357T***	*helicase* (ORF77); 1 synonymous substitution
***A72799G***	ORF81; 1 nonsynonymous substitution
***A73652G***; ***T73660G***	*lef-4* (ORF82); 1 synonymous and 1 nonsynonymous substitutions
Δ76289–76310	ORF85; 1 22-nt deletion of a repeated sequence at the 3’ end of the ORF-no impact on encoded amino acid sequence.
A77453C	ORF86; 1 synonymous substitution
C79226T; G79375A	Between *hr5* and ORF89
A80342G	ORF90; 1 nonsynonymous substitution
***T82426A***; ***G82575A***	*vlf-1* (ORF93); 1 synonymous and 1 nonsynonymous substitutions
***G83369A***	ORF95; 1 nonsynonymous substitution
***A86263G***	*dnapol* (ORF96); 1 synonymous substitution
***A86772G***; ***A87134G***; C88503A; ***C88667A***	*desmoplakin* (ORF97); 1 synonymous and 3 nonsynonymous substitutions
T88858C; T88861C	Between *desmoplakin* and *lef-3*
***G89257A***	*lef-3* (ORF98); 1 synonymous substitution
***C90715T***	ORF100; 1 synonymous substitution
***T91296C***	*iap-5* (ORF101); 1 synonymous substitution
***G92715A***; ***G92800A***; ***C92804A***; C93097A	*lef-9* (ORF102); 1 synonymous and 3 nonsynonymous substitutions
(T) inserted after 93363 (6T to 7T)	Between ORF102 and ORF103
Δ96280 (5T to 4T)	*hr6*

^a^Nucleotide positions in the genome sequence are indicated. For substitutions, the nucleotide identity in the genome followed by the nucleotide position and the nucleotide identity in the associated transcriptomic contig. Deletions (Δ) and insertions are described in relation to the consensus genome sequence. Indels and substitutions in bold italics also are present in a minority of the genome sequence 454 reads.

### Gene content

The PiGV genome contained all 37 of the core genes proposed for *Baculoviridae* by Garavaglia et al. [41](Fig 1). Several non-core genes conserved among all members of *Alphabaculovirus* and *Betabaculovirus* were also found in the PiGV genome, including *granulin*, *pk-1*, *ac13*, *ac23*, *dbp*, *lef-6*, *ubiquitin*, *pp31*, *lef-11*, *ac38*, *fp25k*, *lef-3*, *ac75*, *tlp*, *p12*, *ac106/107*, *ac108*, *ac110*, *p24*, *pep*, *me53*, *ac145*, *ac146*, and *ie-1*. Two genes claimed by Garavaglia et al. to be conserved among betabaculoviruses, *gp37* and *exon0*, were not found in PiGV. These genes also do not occur in the genomes of SpfrGV-VG008, ErelGV, or in isolates of AgseGV.

**Fig 5:**
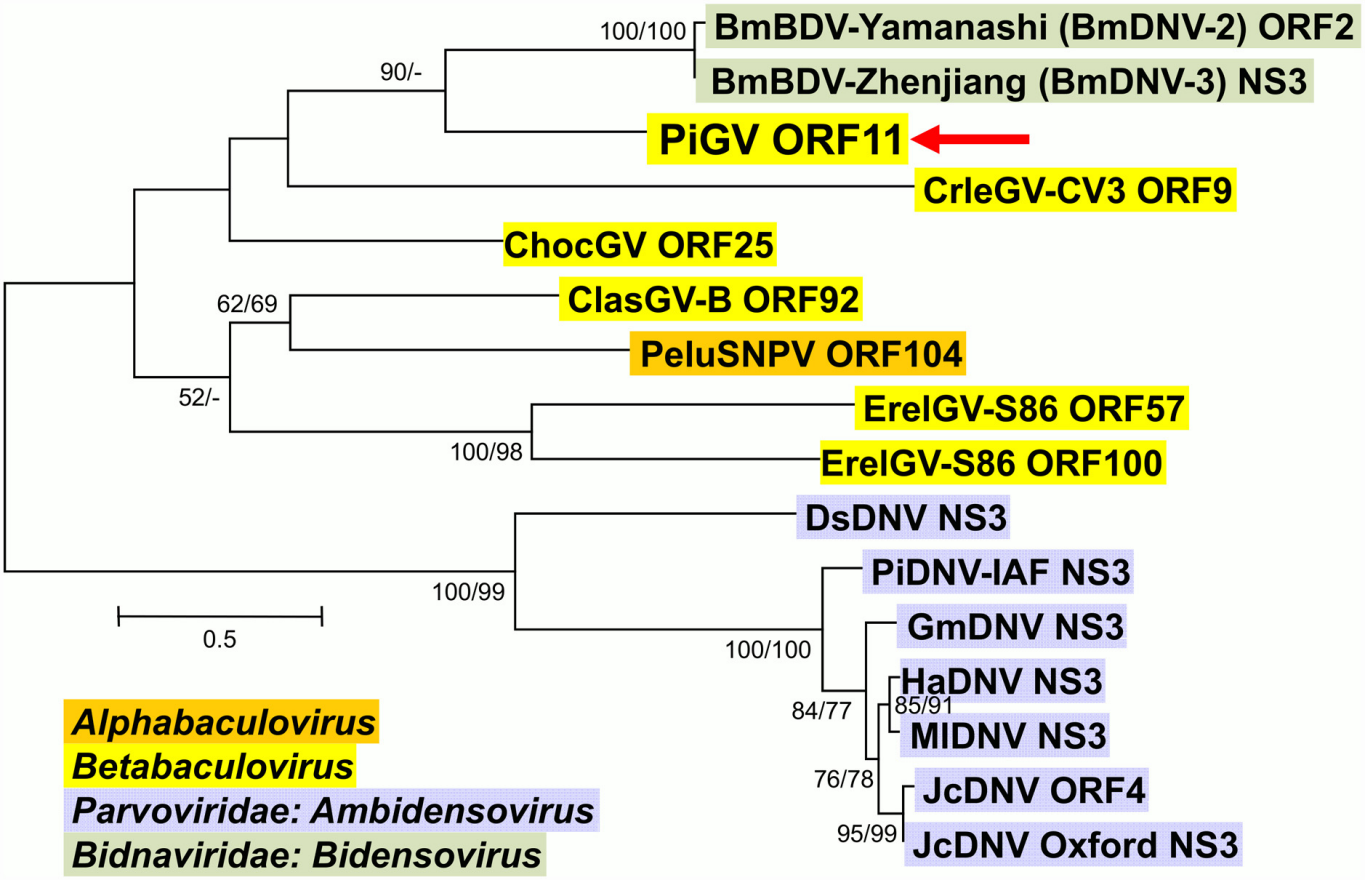
Phylogenetic analysis of baculovirus, bidensovirus, and densovirus NS3 homologues. ME phylogram inferred from the alignment of NS3 homologue amino acid sequences are shown with bootstrap values (>50%) at interior branches for ME and ML analysis (ME/ML) where they occur. In addition to PiGV (indicated by a red arrow), virus taxa are as indicated in S1 Table. Family and/or genus of each taxon is indicated with a color-coded text background.

The remaining ORFs in the PiGV genome consist of (a) ORFs with homologues among granuloviruses (S2 Table) and (b) ORFs with no granulovirus homologues (Table 2). Among the former class of ORFs is PiGV ORF11, which had the most significant blastp match with an ORF from isolates of *Bombyx mori bidensovirus*, the sole species of the recently-created virus family *Bidnaviridae* [54]. Other homologues have been described from a handful of granulovirus genomes, including those of CrleGV-CV3, ChocGV, Clostera anastomosis granulovirus B (ClasGV-B), and ErelGV. A homologue has also been identified in the genome of Perigonia lusca single nucleopolyhedrovirus (PeluSNPV). The encoded amino acid sequences of these genes also share similarity with amino acid sequences of the NS3 gene found in isolates of the densovirus species *Lepidopteran ambidensovirus 1*. Phylogenetic inference of these sequences grouped the densovirus sequences together as well as the two copies of the homologue found in ErelGV with strong bootstrap support (Fig 5). PiGV ORF11 was grouped with the bidensovirus sequences, and homologues from ClasGV-B and PeluSNPV were grouped together with moderate bootstrap support, while there was no significant bootstrap support for the positions of the other homologues. From the phylogeny, it appears that homologues of the densovirus/bidensovirus NS3 sequences appeared in baculovirus genomes as a result of independent acquisition events. This conclusion is consistent with that drawn by Ardisson-Araújo et al. [55] from their analysis of a smaller data set. An alternative hypothesis for the presence of NS3 homologues in the betabaculovirus genomes involves a single ancient acquisition event followed by sequence divergence. The NS3 homologue in PeluSNPV may be due to horizontal gene transmission from a granulovirus, or it may point to an NS3 gene acquisition that predated the divergence of alphabaculoviruses and betabaculoviruses. In either case, for this hypothesis, one would also have to stipulate that the NS3 gene was subsequently lost from most baculovirus lineages. With regard to the possible functional significance of these genes, Junonia coenia densovirus NS3 was shown to be required for viral DNA replication [56], while BmDNV-Zhenjiang NS3 was shown to interact with a transgelin homologue and a serine protein precursor in *B*. *mori* BmN cells [57]. It is unclear what role, if any, the baculovirus homologues of these genes possess in the baculovirus life cycle.

**Table 2:** PiGV ORFs with no orthologues in other granulovirus genomes.

ORF	Position/Size (aa)	Best blastp match	Notes
9 (*dut*)	5169→5594/141	Bathycoccus sp. RCC1105 virus BpV1 dUTPase; 42.6% (60/141)	Blastp match with a single baculovirus *dut* (Spodoptera frugiperda MNPV); no blastp matches to other GV *dut* genes
12	8421←10262/613	-	No conserved domains
15 (*ac11*)	12857←13942/361	Choristoneura fumiferana DEF MNPV ORF9; 21.7% (80/368)	
18 (*ac11*)	15613→16524/303	Bombyx mori NPV-T3 ORF4; 40.4% (130/322)	
22	19988→20560/190	-	No conserved domains
24	20934←21959/341	-	No conserved domains
25	22903→24189/428	-	No conserved domains
28	27464→29122/552	-	Contains SMC_prok_B domain
36	34788←35198/136	-	No conserved domains
40	38516←39430/304	-	No conserved domains
53	49474→50136/330	-	No conserved domains
54	50237→51379/380	-	No conserved domains
81	71674←72873/399	GM24450 [*Drosophila sechellia*]; 30.9% (131/424)	Inhibitor of apoptosis (IAP), with 2 BIR and 1 RING finger domains
87	77672←77974/100	mucin-2-like [*Amyelois transitella*]; 50.0% (34/68)	Many matches with mucin-2 and mucin-2-like sequences; contains ChtBD2 (chitin-binding) domain
103	93414→93659/81	Lymantria dispar MNPV-27 ORF25; 44.3% (35/79)	
105	94221←94562/113	-	No conserved domains

#### ORFs with alphabaculovirus homologues

Four PiGV ORFs–ORF9, ORF15, ORF18, and ORF103 –encode sequences that only exhibit significant sequence similarity to alphabaculovirus ORFs.

ORF9 encodes an amino acid sequence with significant similarity to dUTPases (*dut*) from a number of sources. Although the granuloviruses AgseGV, EpapGV, ErelGV, and SpfrGV-VG008 also encode *dut* homologues, none of these GV dUTPase sequences appeared in a BLASTp search with PiGV ORF9 as a query sequence. The only baculovirus homologue that appeared as a match to ORF9 by blastp was that of Spodoptera frugiperda multiple nucleopolyhedrovirus (SfMNPV). This result suggests that the *dut* homologues in the PiGV genome and other GV genomes are a consequence of a separate gene acquisition events. Recent comprehensive analyses of baculovirus *dut* gene phylogeny [58, 59] supports this hypothesis, in one case finding that the *dut* genes present in baculovirus genomes are the product of at least nine horizontal gene transfer events [59].

**Fig 6:**
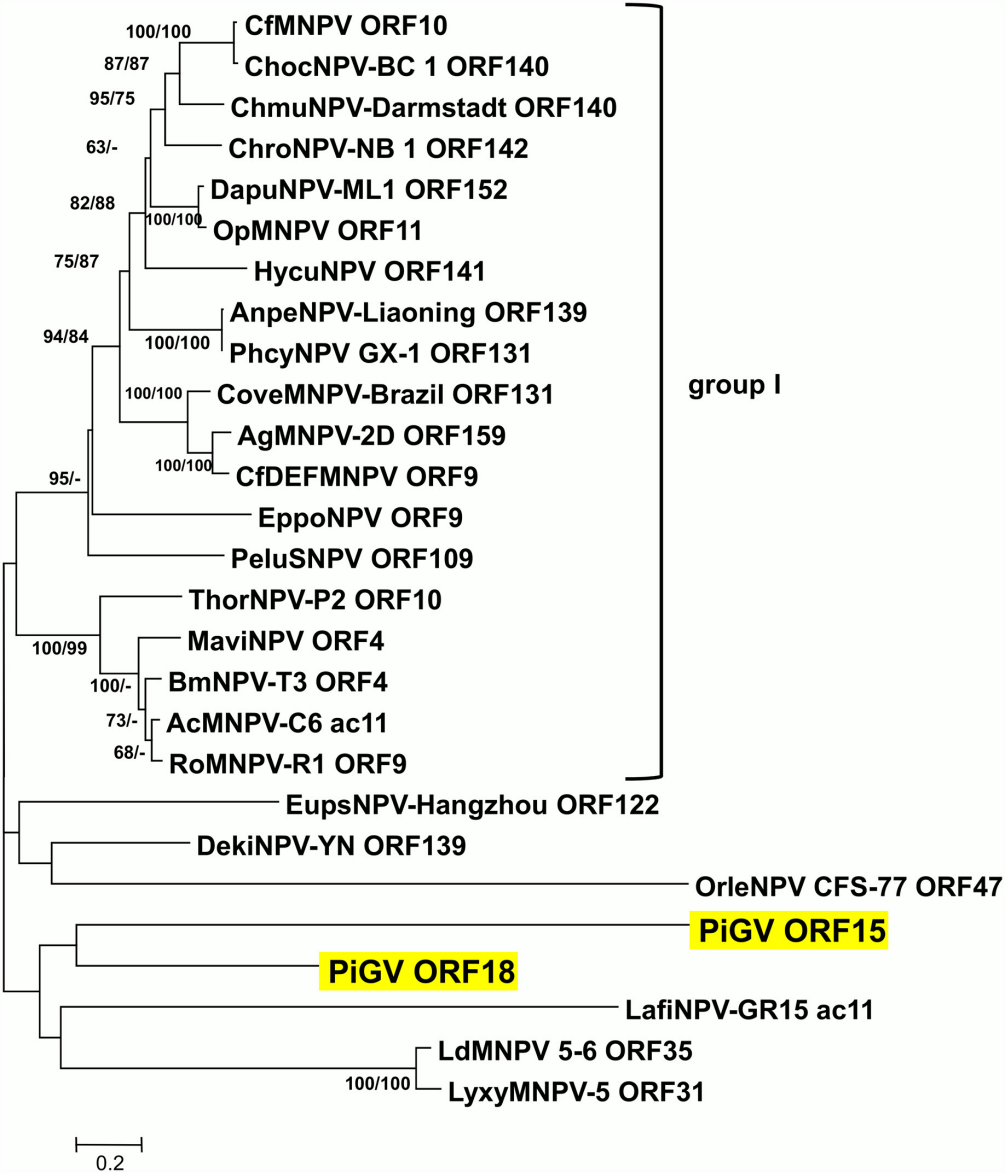
Phylogenetic analysis of baculovirus *ac11* homologues. ME phylogram inferred from the alignment of AcMNPV *ac11* homologue amino acid sequences are shown with bootstrap values (>50%) at interior branches for ME and ML analysis (ME/ML) where they occur. Virus taxa and accession numbers used in the analysis are as indicated in S1 Table. The PiGV *ac11* homologues are highlighted in yellow, and the group I alphabaculovirus sequences are indicated by a bracket.

Both ORF15 and ORF18 encode homologues of the AcMNPV ORF *ac11*. Homologues of this gene have only been found in all group I alphabaculoviruses and a selection of group II alphabaculoviruses. Phylogenetic inference of relationships among *ac11* homologues points to two well-supported clades of group I alphabaculovirus sequences (Fig 6). The homologues from PiGV and the group II alphabaculoviruses occur outside these clades and are separated by long branch lengths, suggesting that these homologues have diverged extensively from each other and/or are the results of horizontal gene transfer. ORF15 and ORF18, which only share 26% amino acid sequence identity with each other, are grouped together but with no significant degree of support. Two homologues of the same gene in the same genome could arise from a single acquisition event followed by gene duplication and sequence divergence. However, given the degree of sequence divergence between the ORF15 and ORF18 sequences, and in the absence of reports of *ac11* homologues in other granulovirus genomes, it seems more likely that ORF15 and ORF18 resulted from independent acquisition events. In cells infected with an AcMNPV *ac11* knockout mutant bacmid-generated virus, both nucleocapsid egress from the nucleus and intranuclear envelopment of nucleocapsids were absent, suggesting an essential role for *ac11* in those particular steps in virion morphogenesis [60]. It is unclear if the PiGV homologues play a similar role in PiGV-infected cells.

Finally PiGV ORF103 encodes a homologue of an ORF annotated in isolates of Lymantria dispar multiple nucleopolyhedrovirus (LdMNPV), Lymantria xylina nucleopolyhedrovirus (LyxyNPV), and isolates of the nudivirus species *Heliothis zea nudivirus*. Although the annotation for the LdMNPV 5–6 isolate genome indicates that this is a homologue of AcMNPV *ac4* [61], blastp and HMMER [62] queries with PiGV ORF103 and the LdMNPV orthologue sequences did not yield matches with *ac4* or any other baculovirus gene.

#### ORFs encoding inhibitor of apoptosis (IAP) homologues

Inhibitors of apoptosis (IAPs) are functionally diverse proteins that were originally identified in baculoviruses as inhibitors of host cell apoptosis [63–67]. They were subsequently found in both eukaryotic organisms and in other DNA viruses. IAPs are recognized by the presence of at least one baculoviral IAP repeat (BIR) domain, which mediates protein interaction. Baculovirus IAPs also contain a copy of a Really Interesting New Gene (RING) domain in the C-terminus.

Clem (2015) [67] identified 6 distinct groups of IAP-encoding genes found in baculovirus genomes. The genes *iap-1*, *iap-2*, and *iap-4* have been found only in alphabaculoviruses, while *iap-5* and *iap-6* genes are found exclusively in betabaculoviruses. Genes of the *iap-3* group are found among alphabaculoviruses, betabaculoviruses, and gammabaculoviruses.

In the PiGV genome, ORF101 was identified as an *iap-5* orthologue. However, no orthologue for *iap-3* or *iap-6* was found. Instead, a second *iap* homologue encoded by PiGV ORF81 was identified that did not appear to correspond to any of the baculovirus *iap* groups described by Clem (2015) [67]. A blastp query with the ORF81 amino acid sequence yielded 189 matching sequences that included mostly IAP sequences from insects of orders Diptera (flies and mosquitoes), Hymenoptera (ants and wasps), Hemiptera (true bugs), with additional matches with a few sequences from Coleoptera (beetles), Isoptera (termites), and Dictyoptera (cockroaches). No baculovirus IAP sequences were among these matches, and only one IAP sequence from a moth, *Galleria mellonella* (order Lepidoptera; Genbank accession no. ACV04797, expected value of 2e^-13^), occurred in the results of the query. *G*. *mellonella*, like *P*. *interpunctella*, is classified in the family Pyralidae. A tBlastn search of the McTaggart et al. [40] *P*. *interpunctella* transcriptome contigs with the sequences of the *G*. *mellonella* IAP and an IAP from the pyralid moth *Amyelois transitella* (Genbank accession no. XP_013192077) failed to yield any matches. A search of the annotation for these contigs also failed to find any sequences identified as encoding inhibitors of apoptosis in the *P*. *interpunctella* transcriptome.

**Fig 7:**
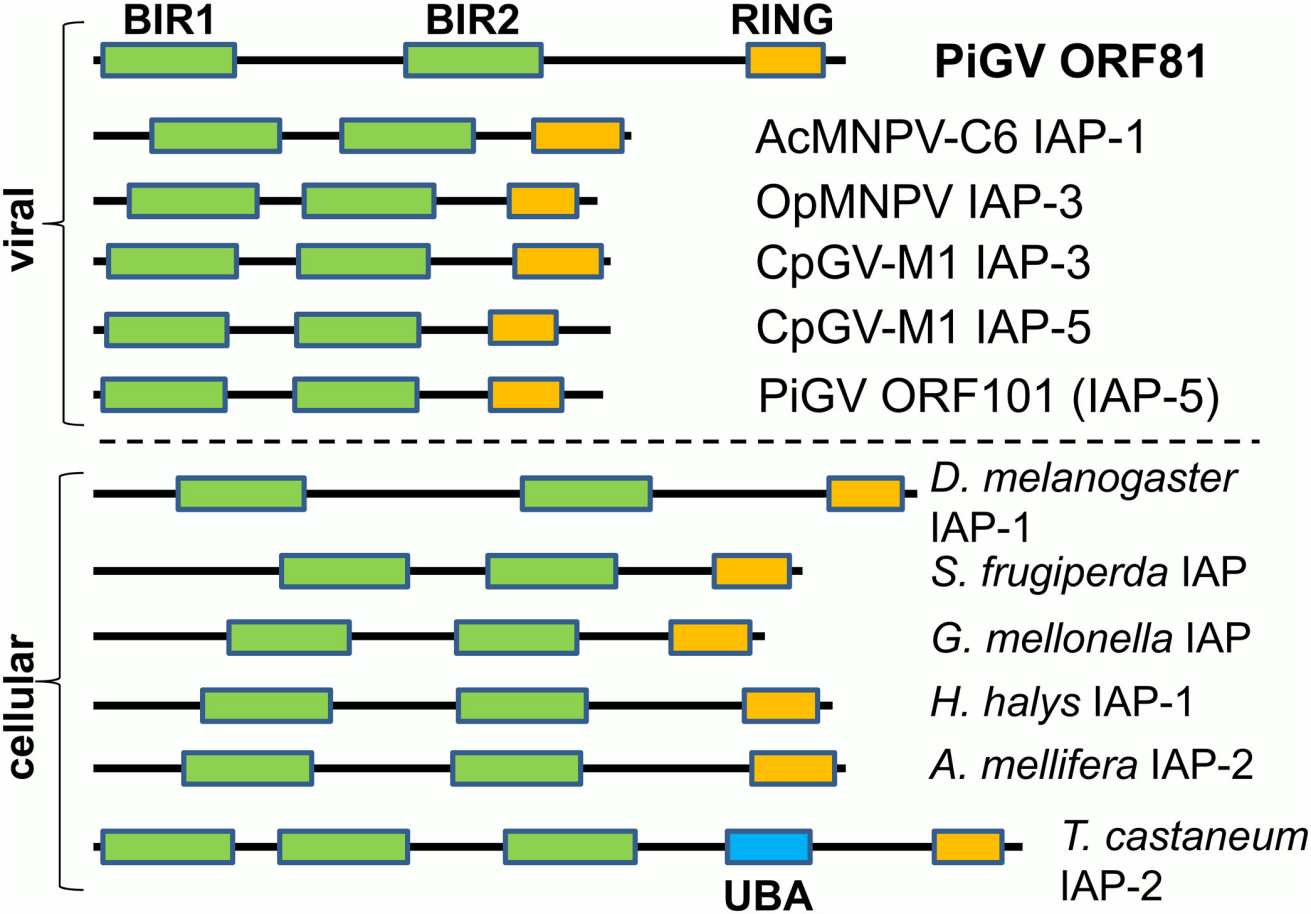
Comparison of PiGV IAPs with representative IAPs from other baculoviruses and insects. A schematic illustration of IAPs from PiGV, selected alphabaculoviruses (AcMNPV-C6, OpMNPV) and betabaculoviruses (CpGV), and insects. Lines representing the IAPs are drawn in proportion to the sizes of the proteins. The BIR domains are shown in green, the RING domain in orange, and the ubiquitin-association domain (UBA) of *T*. *castaneum* IAP-2 is shown in blue.

An examination of the ORF81 amino acid sequence revealed the existence of two BIR motifs and a C-terminal RING domain (Fig 7). ORF81 is thus similar to baculovirus IAP-1, IAP-3, and IAP-5, which also possess two BIRs and one RING domain. However, at 399 amino acids, the protein encoded by ORF81 was significantly larger than those encoded by baculovirus *iap* genes (which range from approximately 240–310 amino acids). Rather, the size of the ORF81 protein was more similar to that of insect cellular IAP homologues (Fig 7). Cellular insect IAPs possess an N-terminal leader sequence occurring prior to the first BIR repeat (BIR1) that is longer than the sequence upstream of BIR1 in baculovirus IAPs (Fig 7). In lepidopteran IAPs, leader sequence contains a mitogen-activated kinase (MAPK) degron motif (TPxxS) which mediates the turnover of cellular IAPs in baculovirus-infected cells [68]. The PiGV ORF81 protein does not possess an extended leader sequence; BIR1 in this protein begins at residue 3, 5, or 7 (depending on the specific conserved domain database that is consulted). Also, no sequences similar to the lepidopteran IAP degron, the caspase cleavage site, or the cellular IAP instability motifs identified by Vandergaast et al (2015) [68] are present in PiGV ORF81.

**Fig 8:**
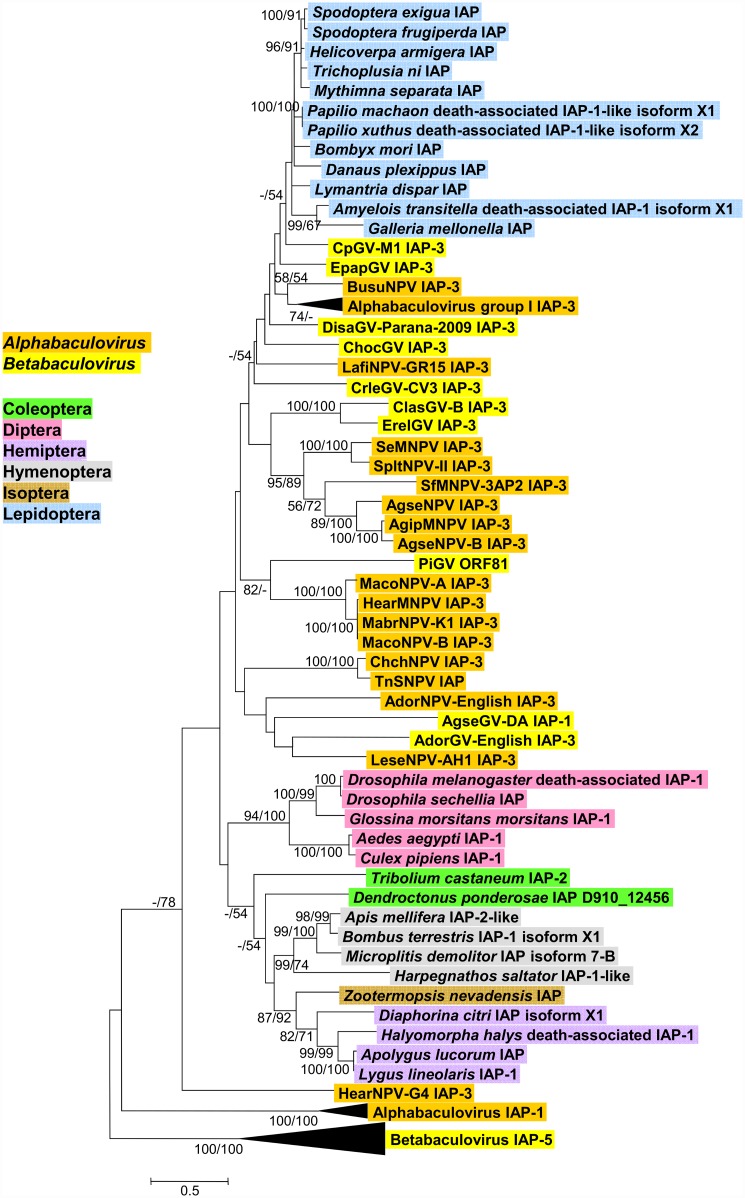
Phylogenetic analysis of viral and cellular IAPs. ML phylogram inferred from the alignment of baculovirus and insect amino acid sequences are shown with bootstrap values (>50%) at interior branches for ME and ML analysis (ME/ML) where they occur. Branches for alphabaculovirus IAP-1, betabaculovirus IAP-5, and group I alphabaculovirus IAP-3 sequences are collapsed, and the nodes for these classes of IAPs are indicated in the tree. The baculovirus genus or insect order for each taxon is indicated with color-coded text background. The virus and insect taxa and their accession numbers are as listed in S1 Table.

Phylogenetic inference was carried out with a selection of cellular IAPs with two copies of the BIR motif that exhibited significant sequence similarity with the PiGV ORF81 product by blastp, along with baculovirus IAPs of the *iap-1*, *iap-3*, and *iap-5* classes (Fig 8). Many of the basal nodes of this tree did not possess ≥50% bootstrap support, but well-supported clades were obtained for the alphabaculovirus IAP-1 and betabaculovirus IAP-5 sequences, as well as sequences from insect orders Diptera, Hymenoptera, Hemiptera, and the moth species of families Noctuidae and Pyralidae. The single termite IAP sequence (from *Zootermopsis nevadensis*) grouped with the hemipteran sequences. While the group I alphabaculovirus IAP-3 sequences were grouped together, the bootstrap support for this clade was not strong. Other IAP-3 sequences from group II alphabaculoviruses and betabaculoviruses did not form a coherent clade, though some IAPs from closely related viruses (e.g. the *Spodoptera* and *Agrotis* spp. NPVs) occurred in terminal and sub-terminal branches that enjoyed good support. The results of this analysis are similar to a comprehensive phylogenetic analysis of IAPs from many different viral and cellular sources conducted by Thézé and coworkers [58], but different from the phylogenetic analysis carried out by Clem (2015) [67] exclusively with viral sequences in which the IAP-3 sequences all occurred in a single clade. PiGV ORF81 was placed in a group with IAP-3 sequences from *Mamestra* spp. NPVs and the IAP-3 from HearMNPV, which is closely related to the *Mamestra* spp. viruses [69]. Both ME and ML methods placed ORF81 in this clade, although the bootstrap value in the ML tree was only 43%. However, ORF81 and the NPV IAP sequences were separated by long branch lengths.

Phylogenetic analysis by Hughes [70] with a smaller set of baculovirus and host insect IAP sequences placed CpGV IAP-3 and four other NPV IAP-3 sequences in a group with three lepidopteran IAPs with a strong degree of support. While CpGV-M1 IAP-3 was also placed with lepidopteran IAPs in our analysis, the relationships of other viral IAP-3 sequences to the lepidopteran sequences is less clear (Fig 8). In addition to using fewer sequences, the analysis of Hughes also employed alignments of the BIR domain sequences only, which may account for some differences in the results. Nevertheless, it seems clear that the baculovirus IAP-1 and IAP-5 lineages are each due to single acquisition events. It is also possible that the baculovirus IAP-3 genes and the PiGV ORF81 IAP homologue are descended from a single ancient acquisition event followed by extensive sequence divergence, although these sequences do not occur in a single well-supported clade in the IAP phylogeny as is the case for the IAP-1 and IAP-5 sequences.

While some baculovirus IAPs of the IAP-3 group possess demonstrated anti-apoptotic activity, not all baculovirus IAPs have been shown to inhibit apoptosis when tested [66]. The IAPs with proven anti-apoptotic activity do not appear to function by directly binding and inhibiting the effector caspases that trigger apoptosis [71, 72], but instead bind to and stabilize cellular IAPs, which are degraded rapidly in response to baculovirus infection [68, 73]. Since both PiGV IAP-5 and ORF81 have short leader sequences with no degron or instability motifs, it is possible that they would inhibit apoptosis by the same mechanism.

## Conclusions

The role of horizontal gene transfer in the evolution and adaptation of organisms has long been recognized [74]. Viruses in particular have been documented as mediators of horizontal gene transfer, and large DNA viruses in particular appear to be mosaics of genes from different sources [75, 76]. In particular, a recent study has provided evidence that baculoviruses may have served to shuttle transposons among different species of host moths [77]. Some environments hypothetically are more conducive to horizontal gene transfer than others. This is likely the case for the Indianmeal moth, *P*. *interpunctella*. As pests of stored grains and nuts in a wide range of climates and locations, larvae of *P*. *interpunctella* conceivably could encounter a wide variety of donor gene sources, including microorganisms. One can imagine opportunities for viruses and other microorganisms resident in *P*. *interpunctella* to acquire genes from a wide taxonomic range of donor genomes. In particular, exposure of *P*. *interpunctella* to the viruses of other insects that infest grains and nuts could conceivably account for the appearance of genes in the PiGV genome that are present in other distinct lineages of viruses, such as alphabaculoviruses and densoviruses. In support of this hypothesis, an analysis of gene homologues shared by baculoviruses and entomopoxviruses found that ORF *xc138*, originally identified in the XecnGV genome, was apparently transferred directly between entomopoxviruses and granuloviruses on two separate occasions [58]. The sequence of PiGV thus serves to further highlight the concept of viruses as agents for horizontal gene transfer, not only from a virus to a host [78], but to other viruses as well.

## Supporting Information

S1 Table Names, abbreviations, and GenBank accession numbers of taxa used in phylogenetic inference. (DOCX)

S2 Table PiGV open reading frames (ORFs) and homologous repeat regions (*hr*s). (DOCX)
