# Supplementary material for: The Complete Genome Sequence of Plodia Interpunctella Granulovirus: Evidence for Horizontal Gene Transfer and Discovery of an Unusual Inhibitor-of-Apoptosis Gene
Source: PLoS One. 2016 Jul 29;11(7):e0160389. doi: 10.1371/journal.pone.0160389 (PMC4966970; doi:10.1371/journal.pone.0160389)
Supplement: S1 Table — (DOCX) [file pone.0160389.s001.docx]

S1 Table. Names, abbreviations, and GenBank accession numbers of taxa used in phylogenetic inference.

| Virus isolate or insect species | Virus abbreviation | Order, family, and/or genus | Genbank accession numbers | | | |
| --- | --- | --- | --- | --- | --- | --- |
|  |  |  | Genome (core genes; Fig 3)^a^ | NS3 homologues (Fig 5) | Ac11 homologues (Fig 6) | IAPs (Fig 8)^b^ |
| Adoxophyes orana nucleopolyhedrovirus English | AdorNPV-English | *Baculoviridae: Alphabaculovirus* |  |  |  | YP_002300603 |
| Agrotis ipsilon multiple nucleopolyhedrovirus | AgipMNPV | *Baculoviridae: Alphabaculovirus* |  |  |  | YP_002268158 |
| Agrotis segetum nucleopolyhedrovirus | AgseNPV | *Baculoviridae: Alphabaculovirus* | DQ123841 |  |  | YP_529789 |
| Agrotis segetum nucleopolyhedrovirus B | AgseNPV-B | *Baculoviridae: Alphabaculovirus* |  |  |  | YP_009112678 |
| Antheraea pernyi nucleopolyhedrovirus Liaoning | AnpeNPV-Liaoning | *Baculoviridae: Alphabaculovirus* |  |  | YP_611103 |  |
| Anticarsia gemmatalis multiple nucleopolyhedrovirus 2D | AgMNPV-2D | *Baculoviridae: Alphabaculovirus* | DQ813662 |  | YP_803404 | YP_803434 (IAP-1), YP_803428 (IAP-3) |
| Autographa californica multiple nucleopolyhedrovirus C6 | AcMNPV-C6 | *Baculoviridae: Alphabaculovirus* | L22858 |  | NP_054040 | NP_054056 (IAP-1) |
| Bombyx mori nucleopolyhedrovirus T3 | BmNPV-T3 | *Baculoviridae: Alphabaculovirus* | L33180 |  | NP_047417 |  |
| Buzura suppressaria nucleopolyhedrovirus | BusuNPV | *Baculoviridae: Alphabaculovirus* |  |  |  | YP_009001870 |
| Choristoneura fumiferana multiple nucleopolyhedrovirus | CfMNPV | *Baculoviridae: Alphabaculovirus* |  |  | NP_848322 | NP_848348 (IAP-1); NP_848342 (IAP-3) |
| Choristoneura fumiferana DEF multiple nucleopolyhedrovirus | CfDEFMNPV | *Baculoviridae: Alphabaculovirus* |  |  | NP_932618 | NP_932645 (IAP-1); NP_932639 (IAP-3) |
| Choristoneura murinana nucleopolyhedrovirus Darmstadt | ChmuNPV-Darmstadt | *Baculoviridae: Alphabaculovirus* |  |  | YP_008992232 | YP_008992207 (IAP-3) |
| Choristoneura occidentalis nucleopolyhedrovirus BC1 | ChocNPV-BC1 | *Baculoviridae: Alphabaculovirus* |  |  | YP_008378642 | YP_008378614 (IAP-1); YP_008378620 (IAP-3) |
| Choristoneura rosaceana nucleopolyhedrovirus NB1 | ChroNPV-NB1 | *Baculoviridae: Alphabaculovirus* |  |  | YP_008378495 |  |
| Chrysodeixis chalcites nucleopolyhedrovirus | ChchNPV | *Baculoviridae: Alphabaculovirus* |  |  | YP_249643 |  |
| Condylorrhiza vestigialis multiple nuclepolyhedrovirus Brazil | CoveMNPV-Brazil | *Baculoviridae: Alphabaculovirus* |  |  | YP_009118614 |  |
| Dasychira pudibunda nucleopolyhedrovirus ML1 | DapuNPV-ML1 | *Baculoviridae: Alphabaculovirus* |  |  | AKR14227 |  |
| Dendrolimus kikuchii nucleopolyhedrovirus YN | DekiNPV-YN | *Baculoviridae: Alphabaculovirus* |  |  | AFS52016 |  |
| Epiphyas postvittana nucleopolyhedrovirus | EppoNPV | *Baculoviridae: Alphabaculovirus* |  |  | NP_203177 | NP_203202 (IAP-1); NP_203195 (IAP-3) |
| Euproctis pseudoconspersa nucleopolyhedrovirus Hangzhou | EupsNPV-Hangzhou | *Baculoviridae: Alphabaculovirus* |  |  | YP_002854732 |  |
| Helicoverpa armigera nucleopolyhedrovirus G4 | HearSNPV-G4 | *Baculoviridae: Alphabaculovirus* | AF271059 |  |  | YP_002332667 |
| Hyphantria cunea nucleopolyhedrovirus | HycuNPV | *Baculoviridae: Alphabaculovirus* |  |  | YP_473329 | YP_473302 (IAP-1); YP_473308 (IAP-3) |
| Lambdina fiscellaria nucleopolyhedrovirus GR15 | LafiNPV-GR15 | *Baculoviridae: Alphabaculovirus* |  |  | YP_009133225 | YP_009133248 |
| Leucania separata nucleopolyhedrovirus AH1 | LeseNPV-AH1 | *Baculoviridae: Alphabaculovirus* |  |  |  | YP_758337 |
| Lymantria dispar multiple nucleopolyhedrovirus 5-6 | LdMNPV 5-6 | *Baculoviridae: Alphabaculovirus* | AF081810 |  | NP_047671 |  |
| Lymantria xylina multiple nucleopolyhedrovirus 5 | LyxyMNPV-5 | *Baculoviridae: Alphabaculovirus* |  |  | YP_003517771 |  |
| Mamestra brassicae nucleopolyhedrovirus K1 | MabrNPV-K1 | *Baculoviridae: Alphabaculovirus* |  |  |  | YP_009011194 |
| Mamestra configurata nucleopolyhedrovirus A 90/2 | MacoNPV-A 90/2 | *Baculoviridae: Alphabaculovirus* | U59461 |  |  | NP_613222 |
| Mamestra configurata nucleopolyhedrovirus B | MacoNPV-B | *Baculoviridae: Alphabaculovirus* |  |  |  | NP_689312 |
| Maruca vitrata nucleopolyhedrovirus | MaviNPV | *Baculoviridae: Alphabaculovirus* |  |  | YP_950734 | YP_950748 (IAP-1) |
| Orgyia leucostigma nucleopolyhedrovirus CFS-77 | OrleNPV CFS-77 | *Baculoviridae: Alphabaculovirus* |  |  | YP_001650957 |  |
| Orgyia pseudotsugata multiple nucleopolyhedrovirus | OpMNPV | *Baculoviridae: Alphabaculovirus* | U75930 |  | NP_046167 | NP_046197 (IAP-1); NP_046191 (IAP-3) |
| Perigonia lusca single nucleopolyhedrovirus | PeluSNPV | *Baculoviridae: Alphabaculovirus* |  | YP_009165704 | YP_009165709 |  |
| Philosamia cynthia nucleopolyhedrovirus GX-1 | PhcyNPV GX-1 | *Baculoviridae: Alphabaculovirus* |  |  | AFY62937 |  |
| Rachiplusia ou multiple nucleopolyhedrovirus R1 | RoMNPV-R1 | *Baculoviridae: Alphabaculovirus* |  |  | NP_703001 |  |
| Spodoptera exigua multiple nucleopolyhedrovirus US | SeMNPV-US | *Baculoviridae: Alphabaculovirus* | AF169823 |  |  | NP_037870 |
| Spodoptera frugiperda multiple nucleopolyhedrovirus 3AP2 | SfMNPV-3AP2 | *Baculoviridae: Alphabaculovirus* |  |  |  | YP_001036403 |
| Spodoptera litura nucleopolyhedrovirus G2 | SpltNPV-G2 | *Baculoviridae: Alphabaculovirus* | AF325155 |  |  |  |
| Spodoptera litura nucleopolyhedrovirus II | SpltNPV-II |  |  |  |  | YP_002332813 |
| Thysanoplusia orichalcea nucleopolyhedrovirus P2 | ThorNPV-P2 | *Baculoviridae: Alphabaculovirus* |  |  | YP_007250422 |  |
| Trichoplusia ni single nucleopolyhedrovirus | TnSNPV | *Baculoviridae: Alphabaculovirus* | DQ017380 |  |  | ACD84806 |
| Adoxophyes orana granulovirus English | AdorGV-English | *Baculoviridae: Betabaculovirus* | AF547984 |  |  | NP_872543; NP_872553 (IAP-5) |
| Agrotis segetum granulovirus DA | AgseGV-DA | *Baculoviridae: Betabaculovirus* | KR584663 |  |  | AKN63337; AKN63394 (IAP-5) |
| Choristoneura occidentalis granulovirus | ChocGV | *Baculoviridae: Betabaculovirus* | DQ333351 | YP_654446 |  | YP_654505; YP_654516 (IAP-5) |
| Clostera anachoreta granulovirus HBHN | ClanGV-HBHN | *Baculoviridae: Betabaculovirus* | HQ116624 |  |  | YP_004376308 (IAP-5) |
| Clostera anastomosis granulovirus A | CalGV-Henan, or ClasGV-A | *Baculoviridae: Betabaculovirus* | KC179784 |  |  | YP_008720048 (IAP-5) |
| Clostera anastomosis granulovirus B | ClasGV-B | *Baculoviridae: Betabaculovirus* | KR091910 | AKS25435 |  | AKS25432 |
| Cryptophlebia leucotreta granulovirus CV3 | CrleGV-CV3 | *Baculoviridae: Betabaculovirus* | AY229987 | NP_891857 |  | NP_891863; NP_891953 (IAP-5) |
| Cydia pomonella granulovirus M1 | CpGV-M1 | *Baculoviridae: Betabaculovirus* | U53466 |  |  | NP_148801; NP_148900 (IAP-5) |
| Diatraea saccharalis granulovirus Parana-2009 | DisaGV-Parana-2009 |  |  |  |  | YP_009182263;  YP_009182301 (IAP-5) |
| Epinotia aporema granulovirus | EpapGV | *Baculoviridae: Betabaculovirus* | JN408834 |  |  | YP_006908519; YP_006908619 (IAP-5) |
| Erinnyis ello granulovirus | ErelGV | *Baculoviridae: Betabaculovirus* | KJ406702 | YP_009091896; YP_009091939 |  | YP_009091936; YP_009091950 (IAP-5) |
| Helicoverpa armigera granulovirus | HearGV | *Baculoviridae: Betabaculovirus* | EU255577 |  |  | YP_001649121 (IAP-5) |
| Phthorimaea operculella granulovirus | PhopGV | *Baculoviridae: Betabaculovirus* | AF499596 |  |  |  |
| Pieris rapae granulovirus Wuhan | PiraGV-Wuhan | *Baculoviridae: Betabaculovirus* | GQ884143 |  |  | YP_003429422 (IAP-5) |
| Plutella xylostella granulovirus K1 | PlxyGV-K1 | *Baculoviridae: Betabaculovirus* | AF270937 |  |  | NP_068317 (IAP-5) |
| Pseudaletia unipuncta granulovirus H | PsunGV-H | *Baculoviridae: Betabaculovirus* | EU678671 |  |  | YP_003422482 (IAP-5) |
| Spodoptera frugiperda granulovirus VG008 | SpfrGV-VG008 | *Baculoviridae: Betabaculovirus* | KM371112 |  |  | YP_009121899 (IAP-5) |
| Spodoptera litura granulovirus K1 | SpltGV-K1 | *Baculoviridae: Betabaculovirus* | DQ288858 |  |  | YP_001257057 (IAP-5) |
| Trichoplusia ni granulovirus | TnGV |  |  |  |  | AF079223_2 (IAP-5) |
| Xestia c-nigrum granulovirus | XecnGV | *Baculoviridae: Betabaculovirus* | AF162221 |  |  | NP_059285 (IAP-5) |
| Neodiprion abietis nucleopolyhedrovirus | NeabNPV | *Baculoviridae: Gammabaculovirus* | DQ317692 |  |  |  |
| Neodiprion lecontei nucleopolyhedrovirus | NeleNPV | *Baculoviridae: Gammabaculovirus* | AY349019 |  |  |  |
| Neodiprion sertifer nucleopolyhedrovirus | NeseNPV | *Baculoviridae: Gammabaculovirus* | AY430810 |  |  |  |
| Culex nigripalpus nucleopolyhedrovirus Florida 1997 | CuniNPV-Florida 1997 | *Baculoviridae: Deltabaculovirus* | AF403738 |  |  |  |
| Bombyx mori bidensovirus Yamanashi | BmDNV-2/ BmBDV-Yamanashi | *Bidnaviridae: Bidensovirus* |  | AAC60741 |  |  |
| Bombyx mori bidensovirus Zhenjiang | BmDNV-3/ BmBDV-Zhenjiang | *Bidnaviridae: Bidensovirus* |  | YP_007714627 |  |  |
| Diatraea saccharalis densovirus | DsDNV | *Parvoviridae: Ambidensovirus* |  | NP_046812 |  |  |
| Galleria mellonella densovirus | GmDNV | *Parvoviridae: Ambidensovirus* |  | NP_899649 |  |  |
| Helicoverpa armigera densovirus | HaDNV | *Parvoviridae: Ambidensovirus* |  | AFK91982 |  |  |
| Junonia coenia densovirus | JcDNV | *Parvoviridae: Ambidensovirus* |  | NP_694826 |  |  |
| Junonia coenia densovirus Oxford | JcDNV Oxford | *Parvoviridae: Ambidensovirus* |  | AGO32182 |  |  |
| Mythimna loreyi densovirus | MlDNV | *Parvoviridae: Ambidensovirus* |  | NP_958098 |  |  |
| Pseudoplusia includens densovirus IAF | PiDNV-IAF | *Parvoviridae: Ambidensovirus* |  | YP_007003822 |  |  |
| *Dendroctonus ponderosae* |  | Coleoptera: Curculionidae |  |  |  | ERL95188 |
| *Tribolium castaneum* |  | Coleoptera: Tenebrionidae |  |  |  | NP_001280519 |
| *Aedes aegypti* |  | Diptera: Culicidae |  |  |  | ABK01289 |
| *Culex pipiens* |  | Diptera: Culicidae |  |  |  | ABP35673 |
| *Drosophila melanogaster* |  | Diptera: Drosophilidae |  |  |  | NP_524101 |
| *Drosophila sechellia* |  | Diptera: Drosophilidae |  |  |  | XP_002030676 |
| *Glossina morsitans morsitans* |  | Diptera: Glossinidae |  |  |  | ABC25070 |
| *Apolygus lucorum* |  | Hemiptera: Miridae |  |  |  | ALB25537 |
| *Diaphorina citri* |  | Hemiptera: Psyllidae |  |  |  | XP_008469338 |
| *Halyomorpha halys* |  | Hemiptera: Pentatomidae |  |  |  | XP_014290877 |
| *Lygus lineolaris* |  | Hemiptera: Miridae |  |  |  | ADK56128 |
| *Apis mellifera* |  | Hymenoptera: Apidae |  |  |  | XP_006570777 |
| *Bombus terrestris* |  | Hymenoptera: Apidae |  |  |  | XP_003393060 |
| *Harpegnathos saltator* |  | Hymenoptera: Formicidae |  |  |  | XP_011136251 |
| *Microplitis demolitor* |  | Hymenoptera: Braconidae |  |  |  | XP_008554573 |
| *Zootermopsis nevadensis* |  | Isoptera: Termopsidae |  |  |  | KDR09161 |
| *Amyelois transitella* |  | Lepidoptera: Pyralidae |  |  |  | XP_013192077 |
| *Bombyx mori* |  | Lepidoptera: Bombycidae |  |  |  | NP_001037024 |
| *Danaus plexippus* |  | Lepidoptera: Nymphalidae |  |  |  | EHJ76448 |
| *Galleria mellonella* |  | Lepidoptera: Pyralidae |  |  |  | ACV04797 |
| *Helicoverpa armigera* |  | Lepidoptera: Noctuidae |  |  |  | ADM32901 |
| *Lymantria dispar* |  | Lepidoptera: Erebidae |  |  |  | BAM63312 |
| *Mythimna separata* |  | Lepidoptera: Noctuidae |  |  |  | BAM76810 |
| *Papilio machaon* |  | Lepidoptera: Papilionidae |  |  |  | XP_014362435 |
| *Papilio xuthus* |  | Lepidoptera: Papilionidae |  |  |  | XP_013173299 |
| *Spodoptera exigua* |  | Lepidoptera: Noctuidae |  |  |  | ABA62322 |
| *Spodoptera frugiperda* |  | Lepidoptera: Noctuidae |  |  |  | AAF35285.1 |
| *Trichoplusia ni* |  | Lepidoptera: Noctuidae |  |  |  | AF195528_1 |

^a^Accession numbers for genome sequences are given. Conceptual translations of core gene sequences from these genomes were used for the Fig 3 phylogeny.

^b^Occurrence of individual IAP sequences in monophyletic groups of IAPs (IAP-1, IAP-3, IAP-5) that are represented as collapsed branches in Fig 8 is indicated.
