# Supplementary material for: The Complete Genome Sequence of Plodia Interpunctella Granulovirus: Evidence for Horizontal Gene Transfer and Discovery of an Unusual Inhibitor-of-Apoptosis Gene
Source: PLoS One. 2016 Jul 29;11(7):e0160389. doi: 10.1371/journal.pone.0160389 (PMC4966970; doi:10.1371/journal.pone.0160389)
Supplement: S2 Table — (DOCX) [file pone.0160389.s002.docx]

S2 Table. PiGV open reading frames (ORFs) and homologous repeat regions (*hr*s)

| **ORF** | **Name** | **Position** | **aa (Da)** | **Top baculovirus blastp match** | **% identity (range of alignment)** | **Notes/AcMNPV homologues** |
| --- | --- | --- | --- | --- | --- | --- |
| **1** | *granulin* | 1🡪747 | 248 | CrleGV-CV3 ORF1 | 94.4% (234/248) | *ac8* |
| **2** | *pp78/83; orf1629* | 744🡨1067 | 107 | ChocGV ORF2 | 40.4% (36/89) | *ac9* |
| **3** | *pk-1* | 1009🡪1836 | 275 | PiraGV-Wuhan ORF3 | 60.9% (170/279) | *ac10* |
| **4** |  | 1873🡨2490 | 205 | PhopGV ORF4 | 55.1% (103/187) |  |
| **5** |  | 2429🡪2665 | 78 | PhopGV ORF5 | 59.5% (44/74) |  |
| ***hr1*** |  | 2690-2871 |  |  |  | 3 repeats |
| **6** | *ie-1* | 2869🡨4170 | 433 | ErelGV-S86 ORF8 | 45.3% (180/397) | *ac147* |
| **7** |  | 4198🡪4776 | 192 | PiraGV-K ORF7 | 45.7% (86/188) | *ac146* |
| **8** | *chtb1* | 4802🡨5098 | 98 | PiraGV-K ORF8 | 70.3% (71/101) | *ac145* |
| **9** | *dut* | 5169🡪5594 | 141 |  |  | Bathycoccus sp. RCC1105 virus BpV1 dutpase; 42.6% (60/141) |
| **10** | *chitinase* | 5649🡨7370 | 573 | PiGV CEH-OX-TN73 chitinase | 99.8% (572/573) | *ac126* |
| **11** |  | 7586🡨8263 | 225 | Clostera anastomosis GV ClasGV-B ORF92 | 32.4% (73/225) | Top match is Bombyx mori bidensovirus 2 ORF2, 43.8% (95/217). Also in other densoviruses |
| **12** |  | 8421🡨10262 | 613 | No significant match |  |  |
| **13** | *odv-e18* | 10847🡨11107 | 86 | SpltGV-K1 ORF10 | 61.0% (47/77) | *ac143*; Also good match with CpGV odv-e18 (87.5% (63/72)) |
| **14** | *p49* | 11108🡨12526 | 472 | PiraGV-Wuhan ORF15 | 63.1% (289/458) | *ac142* |
| **15** |  | 12857🡨13942 | 361 | Choristoneura fumiferana DEF MNPV ORF9 | 21.7% (80/368) | *ac11*; no betabaculovirus homologue |
| **16** | *odv-e56* | 14081🡨15145 | 354 | ChocGV ORF14 | 74.9% (259/346) | *ac148* |
| **17** |  | 15338🡪15532 | 64 | EpapGV ORF26 | 41.7% (25/60) |  |
| **18** |  | 15613🡪16524 | 303 | BmNPV-T3 ORF4 | 40.4% (130/322) | *ac11* (2^nd^ copy) |
| **19** | *pep* | 16558🡨17085 | 175 | ErelGV ORF20 | 68.6% (118/172) | *ac131* |
| **20** |  | 17301🡨18416 | 371 | CrleGV-CV3 ORF21 | 29.8% (77/258) | *ac18* |
| **21** | *pep-p10* | 18830🡪19792 | 320 | ErelGV-S86 ORF21 | 62.5% (212/339) |  |
| **22** |  | 19988🡪20560 | 190 | No significant match |  |  |
| **23** | *pep-2* | 20304🡪20726 | 140 | CpGV-I12 ORF23 | 72.9% (97/133) |  |
| ***hr2*** |  | 20784-20843 |  |  |  | 1 repeat |
| **24** |  | 20934🡨21959 | 341 | No significant match |  |  |
| **25** |  | 22903🡪24189 | 428 | No significant match |  |  |
| **26** | *nrk-1* | 24768🡨25343 | 191 | CrleGV-CV3 ORF15 | 33.2% (61/184) |  |
| **27** | *efp; F protein* | 25418🡪27355 | 645 | PiraGV-Wuhan ORF26 | 60.2% (339/563) | *ac23* |
| **28** |  | 27464🡪29122 | 552 | No significant match |  |  |
| **29** |  | 29534🡨30265 | 243 | ChocGV ORF24 | 32.9% (84/255) |  |
| **30** |  | 30293🡨30922 | 309 | CpGV-M ORF34 | 55.2% (111/201) |  |
| **31** | *pif-3* | 30825🡪31379 | 184 | PiraGV-Wuhan ORF30 | 57.2% (107/187) | *ac115* |
| **32** | *odv-e66* | 31373🡨33550 | 725 | CpGV-I07 ORF37 | 64.9% (408/629) | *ac46* |
| **33** |  | 33588🡪33902 | 104 | CrleGV-CV3 ORF36 | 78.8% (82/104) |  |
| **34** | *lef-2* | 33956🡪34474 | 172 | PhopGV ORF37 | 46.8% (81/173) | *ac6* |
| **35** |  | 34477🡪34722 | 81 | ChocGV ORF30 | 45.7% (37/81) |  |
| **36** |  | 34788🡨35198 | 136 | No significant match |  |  |
| **37** |  | 35251🡨35721 | 156 | Clostera anastomosis GV (ClasGV-B) ORF33 | 39.6% (65/164) |  |
| **38** | *mmp* | 35765🡨37627 | 620 | CrleGV-CV3 ORF43 | 28.4% (147/517) |  |
| **39** | *p13* | 37598🡪38461 | 287 | ChocGV ORF34 | 64.1% (173/270) |  |
| **40** |  | 38516🡨39430 | 304 | No significant match |  |  |
| **41** | *pif-2* | 39451🡪40581 | 376 | ClasGV-B ORF48 | 70.1% (260/371) | *ac22* |
| **42** |  | 40570🡨40779 | 69 | PiraGV-Wuhan ORF41 | 41.5% (27/65) |  |
| **43** |  | 40792🡪43338 | 848 | CpGV-M ORF50 | 30.1% (139/462) |  |
| **44** |  | 43322🡨43936 | 204 | ChocGV ORF37 | 70.8% (143/202) | *ac106/107* |
| **45** |  | 43942🡪44097 | 51 | CpGV-M ORF53 | 70.2% (33/47) | *ac110* |
| **46** | *v-ubi* | 44101🡨44337 | 78 | SfGV-VG008 ORF42 | 89.7% (70/78) | *ac35* |
| **47** | *odv-ec43* | 44371🡪45471 | 366 | PiraGV-Wuhan ORF46 | 64.8% (230/355) | *ac109* |
| **48** |  | 45475🡪45684 | 69 | CpGV-M1 ORF56 | 62.1% (36/58) | *ac108* |
| **49** | *pp31/39k* | 45697🡨46455 | 252 | PiraGV-Wuhan ORF48 | 49.2% (130/264) | *ac36* |
| **50** | *lef-11* | 46439🡨46723 | 94 | ClanGV-HBHN ORF47 | 56.4% (53/94) | *ac37* |
| **51** | *sod* | 46717🡨47232 | 171 | ClasGV-B ORF47 | 69.0% (107/155) | *ac31* |
| ***hr3*** |  | 47277-47402 |  |  |  | 2 repeats |
| **52** | *p74* | 47375🡨49393 | 672 | CpGV-M1 ORF60 | 63.3% (432/683) | *ac138* |
| **53** |  | 49474🡪50136 | 330 | No significant match |  |  |
| **54** |  | 50237🡪51379 | 380 | No significant match |  |  |
| ***hr4*** |  | 51425-51662 |  |  |  | 3 repeats |
| **55** |  | 51712🡨52284 | 190 | ClasGV-B ORF53 | 64.5% (49/76) |  |
| **56** | *p47* | 52302🡪53462 | 386 | PiraGV-K ORF56 | 66.2% (255/385) | *ac40* |
| **57** | *bv-e31* (nudix hydrolase) | 53459🡪54166 | 235 | CrleGV-CV3 ORF62 | 65.7% (140/213) | *ac38* |
| **58** | *p24* | 54175🡪54669 | 164 | PiraGV-E3 ORF61 | 62.6% (102/163) | *ac129* |
| **59** |  | 54700🡪54960 | 86 | CaLGV-Henan ORF60 | 33.7% (31/92) |  |
| **60** | *38.7 kDa* | 55035🡨55517 | 160 | PiraGV-Wuhan ORF59 | 42.1% (75/178) | *ac13* |
| **61** | *lef-1* | 55498🡨56205 | 235 | CpGV-S ORF74 | 59.3% (140/236) | *ac14* |
| **62** | *pif-1* | 55960🡪57825 | 621 | CpGV-M1 ORF75 | 63.0% (331/525) | *ac119* |
| **63** | *fgf* | 57901🡨58548 | 215 | CrleGV-CV3 ORF68 | 33.3% (68/204) | *ac32* |
| **64** |  | 58595🡨58891 | 98 | CaLGV-Henan ORF65 | 37.0% (30/81) |  |
| **65** | *chtb2* | 58969🡪59244 | 91 | CpGV-I07 ORF79 | 37.3% (31/83) |  |
| **66** | *lef-6* | 59204🡨59521 | 105 | PhopGV ORF72 | 46.5% (47/101) | *ac28* |
| **67** | *dbp* | 59573🡨60418 | 281 | PiraGV-Wuhan ORF66 | 41.3% (111/269) | *ac25* |
| **68** |  | 60457🡨60684 | 75 | PiraGV-E3 ORF70 | 56.7% (38/67) |  |
| **69** |  | 60617🡨61291 | 224 | ErelGV-S86 ORF75 | 37.7% (72/191) |  |
| **70** | *p45/p48* | 61214🡪62386 | 390 | PiraGV-Wuhan ORF68 | 74.4% (287/386) | *ac103* |
| **71** | *p12* | 62411🡪62743 | 110 | PiraGV-Wuhan ORF69 | 57.7% (60/104) | *ac102* |
| **72** | *p40; bv/odv-c42* | 62865🡪63989 | 374 | PiraGV-Wuhan ORF70 | 60.0% (216/360) | *ac101* |
| **73** | *p6.9* | 64013🡪64186 | 57 | No matches |  | Annotated as *p6.9* (*ac100*) due to Arg/Ser-rich sequence and conserved position upstream of *lef-5* |
| **74** | *lef-5* | 64223🡨65161 | 312 | CpGV-I07 ORF87 | 61.6% (149/242) | *ac99* |
| **75** | *38k* | 64910🡪65845 | 311 | PiraGV-Wuhan ORF73 | 65.1% (192/295) | *ac98* |
| **76** | *pif-4; odv-e28; 19k* | 65834🡨66322 | 162 | ClasGV-B ORF89 | 62.0% (98/158) | *ac96* |
| **77** | *helicase-1* | 66306🡪69644 | 1112 | PiraGV-Wuhan ORF75 | 53.6% (609/1136) | *ac95* |
| **78** | *odv-e25* | 69657🡨70286 | 209 | CpGV-M1 ORF91 | 81.2% (173/213) | *ac94* |
| **79** | *p18* | 70326🡨70823 | 165 | CpGV-S ORF92 | 49.4% (80/162) | *ac93* |
| **80** | *p33* | 70839🡪71591 | 250 | PiraGV-Wuhan ORF78 | 70.4% (176/250) | *ac92* |
| **81** |  | 71674🡨72873 | 399 |  |  | Top matches with insect inhibitors of apoptosis. |
| **82** | *lef-4* | 72932🡨74332 | 466 | CpGV-I07 ORF95 | 54.0% (259/480) | *ac90* |
| **83** | *vp39* | 74370🡪75230 | 286 | PiraGV-Wuhan ORF81 | 56.3% (160/284) | *ac89* |
| **84** | *odv-ec27* | 75277🡪76116 | 279 | PiraGV-Wuhan ORF82 | 56.4% (162/287) | *ac144* |
| **85** |  | 76290🡨77351 | 353 | PiraGV-Wuhan ORF83 | 40.3% (145/360) |  |
| **86** |  | 77436🡪77702 | 88 | PhopGV ORF93 | 42.9% (42/98) |  |
| **87** |  | 77672🡨77974 | 100 |  |  | Matches to mucin-2-like genes in lepidopterans and Mythimna separata entomopoxvirus |
| **88** | *vp91* | 77985🡨79811 | 608 | PiraGV-Wuhan ORF85 | 44.9% (292/651) | *ac83* |
| ***hr5*** |  | 78935-79017 |  |  |  | 1 repeat; located in vp91; positionally conserved in CpGV, CrleGV, XecnGV |
| **89** | *tlp-20* | 79777🡪80172 | 131 | PiraGV-Wuhan ORF86 | 33.2% (61/184) | *ac82* |
| **90** |  | 80138🡪80713 | 191 | CrleGV-CV3 ORF94 | 73.0% (135/185) | *ac81* |
| **91** | *gp41* | 80706🡪81575 | 289 | CpGV-I07 ORF104 | 72.7% (208/286) | *ac80* |
| **92** |  | 81580🡪81849 | 89 | PhopGV ORF98 | 47.7% (42/88) | *ac78* |
| **93** | *vlf-1* | 81806🡪82909 | 367 | PiraGV-Wuhan ORF90 | 74.9% (265/354) | *ac77* |
| **94** |  | 82866🡪83174 | 102 | CpGV-M1 ORF107 | 71.4% (60/84) | *ac76* |
| **95** |  | 83183🡪83629 | 148 | PiraGV-Wuhan ORF92 | 60.4% (90/149) | *ac75* |
| **96** | *dnapol* | 83630🡨86707 | 1025 | PiraGV-K ORF93 | 63.5% (647/1019) | *ac65* |
| **97** | *desmoplakin* | 86676🡪88712 | 678 | CpGV-S ORF112 | 33.1% (174/525) | *ac66* |
| **98** | *lef-3* | 89002🡨90012 | 336 | PiraGV-Wuhan ORF99 | 35.6% (110/309) | *ac67* |
| **99** | *pif-6* | 89951🡪90352 | 133 | ChocGV ORF93 | 44.4% (56/126) | *ac68* |
| **100** |  | 90391🡪90900 | 169 | PiraGV-K ORF97 | 41.1% (67/163) |  |
| **101** | *iap-5* | 90922🡪91734 | 270 | CrleGV-CV3 ORF106 | 57.6% (155/269) |  |
| **102** | *lef-9* | 91724🡪93199 | 491 | PiraGV-Wuhan ORF95 | 75.2% (367/488) | *ac62* |
| **103** |  | 93414🡪93659 | 81 | LdMNPV-27 ORF25 | 44.3% (35/79) |  |
| **104** | *fp25k* | 93770🡪94213 | 173 | PiraGV-Wuhan ORF100 | 68.0% (100/147) | *ac61* |
| **105** |  | 94221🡨94562 | 113 | No significant matches |  |  |
| **106** | *DNA ligase* | 94534🡨96216 | 560 | PiraGV-Wuhan ORF102 | 53.1% (299/563) |  |
| ***hr6*** |  | 96237-96318 |  |  |  | 1 repeat |
| **107** |  | 96380🡪96541 | 53 | ClanGV-HBHN ORF105 | 47.2% (25/53) |  |
| **108** |  | 96638🡪96835 | 65 | ClanGV-HBHN ORF106 | 46.4% (32/69) |  |
| **109** | *fgf-2* | 96880🡨97773 | 297 | CpGV-M1 ORF123 | 29.2% (89/305) |  |
| **110** | *alk-exo* | 98044🡪99216 | 390 | PiraGV-K ORF107 | 59.1% (233/394) | *ac133* |
| **111** | *helicase-2* | 99146🡪100498 | 450 | PiraGV-Wuhan ORF108 | 58.7% (244/416) |  |
| **112** | *rr1* | 100618🡨102438 | 606 | PhopGV ORF119 | 56.7% (351/619) |  |
| **113** | *rr2a* | 102543🡪103598 | 351 | PhopGV ORF120 | 58.8% (201/342) |  |
| **114** | *lef-8* | 103711🡨106311 | 867 | PiraGV-Wuhan ORF110 | 69.9% (616/881) | *ac50* |
| ***hr7*** |  | 105116-105180 |  |  |  | 1 repeat; located in *lef-8*; positionally conserved in CpGV, CrleGV, XecnGV |
| **115** |  | 106750🡨107064 | 104 | AgseGV-XJ ORF120 | 43.4% (23/53) |  |
| **116** |  | 107337🡨107167 | 56 | ClanGV-HBHN ORF114 | 30.8% (16/52) |  |
| **117** |  | 107327🡪107728 | 133 | CpGV-M1 ORF134 | 66.2% (88/133) | *ac53* |
| **118** |  | 107725🡨108597 | 290 | ClasGV-B ORF118 | 28.8% (59/205) |  |
| **119** |  | 108599🡨108805 | 68 | ClanGV-HBHN ORF117 | 36.2% (21/58) |  |
| **120** | *lef-10* | 108786🡪109016 | 76 | PiraGV-E3 ORF120 | 52.0% (39/75) | *ac53a* |
| **121** | *vp1054* | 108880🡪109875 | 331 | PiraGV-Wuhan ORF116 | 59.3% (197/332) | *ac54* |
| **122** | *egt* | 110091🡨111473 | 460 | Lacanobia oleracea granulovirus egt | 53.2% (247/464) | *ac15* |
| **123** | *me53* | 111591🡪112487 | 298 | CpGV-M1 ORF143 | 48.3% (146/302) | *ac139* |
